# Wnt Signaling Across Adult Skin Mini-Organs: Interfollicular Epidermis, Hair Follicle, and Nail—Implications for Disease and Regeneration

**DOI:** 10.3390/ijms27083402

**Published:** 2026-04-10

**Authors:** Anna Pulawska-Czub, Ajay Jakhar, Konrad Łukaszyk, Krzysztof Kobielak

**Affiliations:** 1 Laboratory of Stem Cells, Development and Tissue Regeneration, Centre of New Technologies (CeNT), University of Warsaw (UW), 02-097 Warsaw, Poland; a.pulawska-czub@cent.uw.edu.pl (A.P.-C.); a.jakhar@cent.uw.edu.pl (A.J.); k.lukaszyk@cent.uw.edu.pl (K.Ł.); 2 Faculty of Medicine, University of Warsaw, 02-089 Warsaw, Poland

**Keywords:** Wnt signaling, β-catenin, skin homeostasis and regeneration, hair follicle cycle and regeneration, nail growth and digit regeneration, HFSCs, NSCs, NPFSCs, therapeutic approaches

## Abstract

Skin and its appendages form an integrated system of ectodermal mini-organs that rely on Wnt signaling for lifelong homeostasis and regeneration; yet, the pathway operates in a highly organ-specific manner in each compartment. In interfollicular epidermis, the Wnt activity is spatially graded, thus maintaining the balance between basal progenitor proliferation and terminal differentiation. The hair follicle is governed by an intrinsic oscillator based on cross-regulation between Wnt and BMP signaling, providing a cell-autonomous layer of control over hair cycle dynamics. Finally, the nail organ is characterized by the spatial compartmentalization of Wnt activity, with a distal matrix activation zone supported by specialized mesenchymal niche cells that sustain continuous nail plate growth and coordinate the digit tip regeneration. Understanding these divergent regulatory architectures provides a conceptual framework for targeted regenerative strategies aimed at enhancing repair in skin and its appendages. Therefore, in this review, we synthesize recent molecular studies on Wnt signaling in the adult skin, hair follicles, and nail mini-organs, highlighting appendage-specific features that underlie their distinct regenerative capacities. We further discuss how dysregulated Wnt signaling contributes to skin, hair, and nail pathologies such as alopecia, chronic wounds, excessive scarring, skin cancer, and nail deformations, and summarize the emerging strategies that target Wnt pathway to therapeutically enhance hair regrowth, wound repair, cancer treatment, and digit tip regeneration.

## 1. Introduction

**Figure 1:**
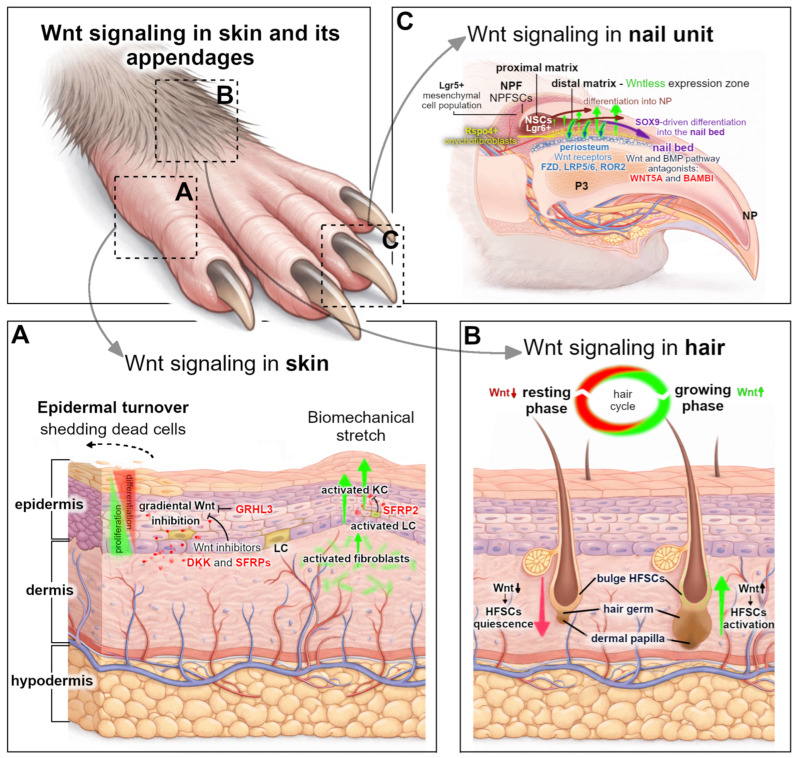
Schematic representation of skin appendages and Wnt signaling dynamics based on the mouse model anatomy. Skin-derived mini-organs—interfollicular epidermis (**A**), hair follicles (**B**), and nails (**C**)—despite sharing a common ectodermal origin, exhibit distinct morphologies and regenerative capacities, with Wnt activity operating in a tightly regulated, organ-specific manner. (**A**) In the epidermis, Wnt ligands modulated β-catenin levels in KC to sustain gradual epidermal turnover, while Wnt inhibitors, such as SFRPs or DKK proteins, along with the transcription factor GRHL3, prevent aberrant stem cell expansion or pathological differentiation. Biomechanical stretch stimulates β-catenin in affected KC and LC, enhancing their proliferation to accommodate tissue expansion. Simultaneously, LC secretes SFRP2, inhibiting intensified Wnt activation in affected skin, thus stimulating KC differentiation and maturation. The activation of Wnt signaling in the deeper dermal layer stimulates fibroblast to proliferate, migrate, and produce more collagen to support structural adaptation of stretched skin. (**B**) Hair follicles are governed by a Wnt-dependent intrinsic oscillator, providing a cell-autonomous layer of control over hair cycle dynamics, responsible for HFSC activation and resting-to-growth phase transition. (**C**) Within the nail mini-organ, Wnt signaling is spatially compartmentalized: A distal matrix Wnt-activation zone (green arrows), characterized by Wntless expression, drives proximally localized Lgr6-positive NSCs to continuously differentiate into the NP (brown arrows). Rspo4-positive onychofibroblasts positioned beneath the nail matrix and nail bed further enhance canonical Wnt signaling in NSCs via R-spondin-Lgr interaction (yellow). Wnt ligands also engage with periosteal Wnt-responsive mesenchymal cells through Wnt receptors (FZD, LRP5/6, and ROR2), supporting distal phalanx (P3) bone growth (blue arrows). In presence of SOX9 transcription factor, NSCs also differentiate into the nail bed (purple arrow), rich in Wnt and Bone morphogenetic protein (BMP) canonical pathway antagonists WNT5A and BAMBI, respectively, suppressing Wnt and BMP activity in this distal digit tip compartment. An additional population of NPFSCs which contribute long-term to the renewal of the peri-nail epidermis is localized in the NPF adjacent to a unique mesenchymal population of Lgr5-expressing cells; however, their interactions and roles in nail homeostasis are not defined. KC—keratinocytes, LC—Langerhans cells, HFSCs—hair follicle stem cells, NSCs—nail stem cells, NPF—nail proximal fold, NPFSCs—nail proximal fold stem cells, NP—nail plate, P3—3rd distal phalanx bone, DKK—Dickkopf proteins, SFRPs—Secreted Frizzled-Related Proteins, GRHL3—Grainyhead Like Transcription Factor 3, Lgr—Leucine-rich repeat-containing G-protein coupled receptors, FZD—Frizzled family receptors, ROR2—Receptor Tyrosine Kinase-like Orphan Receptor 2.

Skin and its appendages, hair follicles, nails, and assorted glands, form a modular system of mini-organs—related but distinct ectodermal structures, each with its own stem cells surrounded by specialized niches, causing them to exhibit strikingly different morphologies and regenerative capacities. They rely on a limited set of conserved signaling pathways, amongst which the tightly controlled wingless-type MMTV integration site family (Wnt) dynamic sustains their lifelong growth and repair [1,2,3,4]. Despite sharing a common molecular framework, Wnt signaling operates in a highly organ-specific and context-dependent manner (Figure 1) [5]. Within the interfollicular epidermis, Wnt signaling regulates the balance between basal stem cell self-renewal and differentiation, sustaining epidermal homeostasis and barrier function, and becomes upregulated at wound edges during wound repair [3,6,7,8]. In the hair follicle (HF), canonical Wnt activation initiates the transition from quiescent telogen to anagen, driving hair follicle stem cell (HFSC) proliferation, while signal suppression contributes to telogen maintenance and pathological outcomes, like androgenetic alopecia [1,9,10,11]. In the nail organ, spatially restricted Wnt activity within the nail distal matrix and surrounding mesenchyme orchestrates continuous nail plate (NP) growth and, uniquely among mammalian appendages, supports digit tip regeneration following shallow amputation [12,13,14]. This mini-organ has emerged as a powerful model for studying Wnt-dependent epithelial–mesenchymal crosstalk, stem cell regulation, and the delicate balance between regeneration and fibrosis within a narrowly defined anatomical window.

The disruption of Wnt signaling homeostasis leads to profound pathological consequences. Insufficient pathway activation impairs wound healing and the regenerative capacity, whereas sustained or ectopic activation promotes fibrosis and tumorigenesis [6,15]. In chronic wounds, the dysregulation of Wnt activity contributes to defective re-epithelialization and angiogenesis [16]. In diabetic wounds, the Wnt pathway shows altered activity, particularly through a decreased R-spondin 3 protein expression, which is an important factor distinguishing chronic, non-healing wounds from normal wound healing processes [17]. Conversely, fibrotic diseases exhibit persistent Wnt activation, driving the fibroblast-to-myofibroblast transition and excessive extracellular matrix deposition, often in cooperation with TGF-β signaling [18]. In skin cancers, Wnt pathway dysregulation supports tumor initiation and progression, and the maintenance of cancer stem cell populations, frequently interacting with other oncogenic networks [19,20]. These divergent outcomes underscore a central principle: Wnt signaling does not function as a simple on/off switch but rather as a graded and context-dependent factor, whose spatial, temporal, and quantitative regulation determines tissue fate. The same pathway that promotes regeneration in one context may drive fibrosis or malignant transformation in another. This duality presents both a challenge and an opportunity for therapeutic intervention.

Rapid progress has recently been achieved in designing therapeutic targeting and modulating Wnt signaling across skin pathologies. Pharmacologic inhibitors, porcupine antagonists, tankyrase inhibitors, and β-catenin transcriptional modulators are being explored for fibrotic diseases and cancers [21,22], while small-molecule activators, regenerative cell therapies, biomaterials, and photobiomodulation approaches aim to restore Wnt activity in hair loss and chronic wounds [23,24,25]. In the nail and digit, the experimental modulation of *β-catenin* and *Lgr6* (Leucine-rich repeat-containing G-protein coupled receptor) has demonstrated the capacity to influence regenerative outcomes, highlighting an untapped translational potential [26]. However, clinical implementation remains constrained by an incomplete mechanistic understanding, pathway crosstalk complexity, safety concerns related to stem cell maintenance, and challenges in achieving precise temporal control.

In this review, we synthesize the current knowledge on Wnt signaling in skin, hair, and nail biology, emphasizing organ-specific regulatory mechanisms, pathological dysregulation, and emerging therapeutic strategies. By integrating insights from regenerative biology, fibrosis, oncology, and appendage research, we aim to delineate how the context-dependent modulation of Wnt signaling can be harnessed to promote tissue repair, while minimizing adverse outcomes.

## 2. Overview of Wnt Signaling Pathways

**Figure 2:**
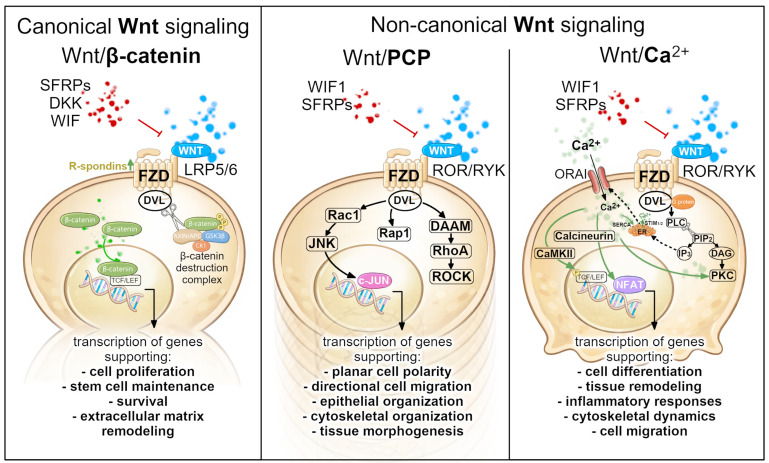
Overview of canonical and non-canonical Wnt signaling pathways. Canonical Wnt signaling (**left** panel) is initiated by binding of Wnt ligands to FZD receptors and LRP5/6 co-receptors at the cell surface. This interaction recruits DVL and inhibits the β-catenin destruction complex. As a result, β-catenin accumulates in the cytoplasm and translocates to the nucleus, where it interacts with TCF/LEF transcription factors to regulate target gene expression involved in proliferation, stem cell maintenance, and extracellular matrix remodeling. Non-canonical Wnt signaling includes the planar cell polarity (PCP) pathway (**middle** panel), which regulates directional cell polarity, cell migration, and epithelial organization through small GTPases such as RhoA (via DAAM1), Rac, and Rap1, as well as downstream effectors including ROCK, JNK, and c-JUN. The Wnt/Ca^2+^ pathway (**right** panel) is initiated by intracellular calcium release following G-protein-mediated activation of PLC, which cleaves PIP_2_ into IP_3_ and DAG. IP_3_ binds to its receptors on the ER membrane, triggering Ca^2+^ release into the cytoplasm. This depletion of ER calcium subsequently induces Ca^2+^ influx, mediated by STIM_1_ protein and ORAI channels, while SERCA pumps contribute to calcium reuptake into the ER. Elevated intracellular Ca^2+^ levels activate calcium-dependent signaling molecules such as PKC (via DAG), CaMKII, and calcineurin-NFAT, thereby regulating cell differentiation, tissue remodeling, migration, and inflammatory responses. Distinct extracellular modulators regulate individual branches of Wnt signaling: canonical Wnt signaling is strongly controlled by antagonists such as DKKs, SFRPs, and WIF1, but also enhanced by R-spondins, whereas non-canonical Wnt pathways are primarily activated by ligands such as WNT5A and WNT11 and are less specifically inhibited by extracellular regulators as WIF1 and SFRPs.

The Wnt signaling network represents one of the most evolutionarily conserved intercellular communication systems in animals regulating crucial processes including embryonic development, stem cell maintenance, cell fate, tissue patterning, polarity, and regeneration. The activation of the Wnt pathway is initiated by a family of secreted glycoproteins, which interact with members of the Frizzled (FZD) receptor family and a range of cell surface co-receptors [27]. However, the outcome of Wnt activation depends on multiple factors, including the combination of Wnt ligands, receptor expression patterns, co-receptors, and intracellular signaling components present within a given cell type [27]. As a result, identical Wnt ligands can activate different downstream pathways depending on the cellular context. Most importantly, upon ligand binding, intracellular signaling cascades activate either canonical (β-catenin-dependent) or non-canonical Wnt signaling branches [28], which share several upstream components but diverge in their intracellular signaling outputs and biological functions (Figure 2).

The canonical Wnt pathway centers on the regulation of the transcriptional co-activator β-catenin. In the absence of Wnt ligands, cytoplasmic β-catenin is continuously degraded by the destruction complex, consisting of the scaffold proteins Axin and APC, together with the glycogen synthase kinase 3 (GSK3β) and casein kinase 1 (CK1), ultimately preventing the activation of Wnt/β-catenin target genes. The activation of canonical Wnt signaling occurs when Wnt ligands bind to a receptor complex composed of FZD receptors and the low-density lipoprotein receptor-related proteins 5/6 (LRP5/6) co-receptors on the cell surface [29]. This interaction recruits the intracellular adaptor protein Dishevelled (DVL) and promotes the assembly of receptor-associated signaling complexes that inhibit the activity of the β-catenin destruction complex. As a consequence, β-catenin accumulates in the cytoplasm and translocates into the nucleus, where it interacts with the TCF/LEF (T-cell factor/lymphoid enhancer binding factor) transcription factors, activating the expression of Wnt target genes involved in proliferation, stem cell maintenance, and differentiation (Figure 2, left panel) [29]. However, canonical Wnt signaling is tightly controlled by extracellular modulations that regulate ligand availability and receptor activation. Secreted inhibitors such as Dickkopf (DKK) proteins, secreted Frizzled-related proteins (SFRPs), and the Wnt inhibitory factor (WIF) attenuate pathway activation, whereas R-spondin (Rspo) proteins enhance signaling by stabilizing FZD receptors at the cell surface. Through this multilayered regulatory system, tissues are able to fine-tune the Wnt activity in a spatially and temporally controlled manner.

Non-canonical Wnt signaling comprises several β-catenin-independent pathways, most prominently the planar cell polarity (PCP) and the Wnt/Ca^2+^ pathways, with additional receptor-specific branches involving receptor tyrosine kinase-like orphan receptors (ROR) and receptor-like tyrosine kinase (RYK) receptors described in certain cellular contexts [30]. The Wnt/PCP pathway regulates the cell polarity and directional cell behavior within the plane of epithelial tissues. This signal is typically activated by Wnt ligands such as WNT5A or WNT11 interacting with FZD receptors and co-receptors including ROR1/2 or RYK. Downstream DVL activates small GTPases RhoA (primarily through the formin-associated protein DAAM1), Rac, and Rap1, which, in turn, regulate the cytoskeletal dynamics and cell polarity through effectors including Rho-associated protein kinase (ROCK) and c-Jun N-terminal kinase (JNK) (Figure 2, middle panel) [31,32,33]. Wnt/PCP signaling therefore plays a crucial role in processes that require coordinated cellular orientation, including epithelial organization, cell migration, and tissue morphogenesis.

In the Wnt/Ca^2+^ signaling pathway, the Wnt ligand binding to the FZD receptor activates G protein working together with Dvl to activate phospholipase C (PLC). Active PLC catalyzes the breakdown of the membrane phospholipid phosphatidylinositol 4,5-bisphosphate (PIP_2_) into two important messengers: inositol triphosphate (IP_3_) and diacylglycerol (DAG). IP_3_ triggers the intracellular calcium release from the endoplasmic reticulum (ER) into cytosol, lowering the calcium level in the endoplasmic reticulum (ER), which is detected by the STIM1 protein (Stromal Interaction Molecule 1). Activated STIM1 translocates to the cell membrane and physically binds and activates ORAI channels, opening them in the plasma membrane, which results in calcium flowing in from the extracellular space, bringing more Ca^2+^ into the cell and back into ER through SERCA pumps. On the other hand, DAG remains in the membrane and participates in the activation of protein kinase C (PKC) along with an increased calcium level, which also stimulates other calcium-sensitive signaling molecules such as calcium/calmodulin-dependent protein kinase II (CaMKII), and calcineurin (Figure 2, right panel). PKC and CaMKII regulate cytoskeletal dynamics and cell migration, with the latter also inhibiting canonical Wnt signaling by promoting the phosphorylation-dependent repression of TCF/LEF transcriptional activity [30]. In parallel, elevated Ca^2+^ levels activate the calcineurin, which enables the translocation of NFAT transcription factors to the nucleus, where they regulate gene expression programs associated with cell differentiation, inflammatory responses, and tissue remodeling [28].

The signaling versatility enables Wnt pathways to control a wide range of biological processes across adult tissues. In the skin, for example, Wnt signaling governs stem cell activation, epithelial differentiation, and tissue regeneration, but its functional implementation differs markedly between skin compartments such as the interfollicular epidermis, hair follicles, and the nail mini-organ. Understanding these context-specific regulatory architectures provides the conceptual foundation for interpreting the organ-specific roles of Wnt signaling as discussed in the following sections.

## 3. Wnt Signaling in Skin

Mammalian skin is composed of three main layers. The first one, the epidermis, gives rise to various appendages, such as hair follicles, sebaceous glands, and sweat glands, which function together as a physical barrier that protects the body from mechanical trauma, pathogens, sunlight burns, and chemical or environmental stress. This avascular structure is nourished by blood vessels and protein fibers in the underlying second layer—dermis. The third layer, the hypodermis, is an adipose tissue that provides energy resources and thermal insulation (Figure 1A).

The integrity of the skin barrier relies on the proper physical, biochemical, and immunological organization of its individual layers. As a result, the skin represents a highly dynamic organ characterized by continuous cellular turnover during routine tissue maintenance as well as in response to injury. This regenerative capacity is sustained by resident stem cell pools that support lifelong epidermal renewal [34]. Wnt signaling is a key regulator of stem cell maintenance and fate determination across multiple tissues and plays a central role in governing skin homeostasis and regeneration.

### 3.1. Wnt Signaling in Skin Homeostasis and Mechanical Stretch

The maintenance of the stratified epidermis depends on a balance between basal progenitor proliferation and the upward differentiation of keratinocytes (KCs) to replace shedding cells. This system is generally maintained at relatively low but spatially regulated levels, reflecting the need to uphold this balance [1,19]. Canonical Wnt activity in epidermal progenitors is associated with the regulation of epidermal stem cell self-renewal and lineage decisions, significantly contributing to the maintenance of basal KC proliferation, thus supporting the epidermal architecture (Figure 1A) [35].

Multiple Wnt ligand coding genes are expressed in the upper layers of skin—epidermis and dermis, including *WNT3*, *WNT4*, *WNT7*, *WNT10A*, and *WNT10B*, which are capable of activating canonical Wnt signaling depending on the receptor context [3]. Epidermal stem and progenitor cells themselves can act as a source of Wnt ligands, forming autocrine or short-range paracrine signaling loops that support basal cell renewal. For example, in mice, KCs have been shown to express *Wnt4* and *Wnt10a* ligands, contributing to the local maintenance of the proliferative capacity [36]. Dermal fibroblasts also express Wnt ligands, providing mesenchymal cues that influence epidermal behavior through epithelial–mesenchymal crosstalk [3]. However, the excessive canonical Wnt activation in the interfollicular epidermis (IFE) can induce hyperproliferation and tumorigenesis; therefore, the pathway output must remain strictly limited by the secretion of Wnt antagonists, which would restrict its activation spatially and temporally. The most prominent inhibitor families include DKK proteins and SFRPs, which act in a paracrine manner to restrain β-catenin activity in basal KCs and thereby maintain the epidermal proliferation–differentiation balance during skin homeostasis [37]. DKK proteins, particularly DKK1 and DKK3, produced primarily by dermal fibroblasts, but also KC and hair follicle (HF) cells, are present in both epidermal and dermal layers, where they inhibit canonical Wnt signaling by binding to LRP5/6 co-receptors and promoting their internalization, thereby preventing Wnt-FZD receptor complex formation [38]. SFRPs are expressed by multiple skin cell types, including dermal fibroblasts, epidermal KCs, or immune cells within the skin tissue, such as dendritic Langerhans cells (LCs) (Figure 1A). SFRPs regulate canonical Wnt signaling by sequestering ligands or interacting with Frizzled receptors, thereby reducing the pathway activity [3]. These antagonists help maintaining epidermal stem cell homeostasis by creating a “Wnt-low” microenvironment, since the moderate activation of Wnt signaling in the IFE leads to the expansion of the stem cell compartment (on the other hand, excessive Wnt activation can redirect interfollicular KCs to adopt HF-like fates rather than normal IFE) [39]. Thus, the suppression of Wnt signaling by antagonists, along with the transcription factor GRHL3 (Grainyhead Like Transcription Factor 3) in IFE stem cells, is important for promoting the differentiation of cells moving beyond the basal layer (Figure 1A). Moreover, the recent discovery of additional transcriptional regulator *Zeb1* demonstrated its cooperation with YAP to activate the *Axin2* expression, and the inhibition of Wnt signaling that maintains both the basal cell fate and stem cell quiescence, and it functions, in part, through suppressing Wnt signaling [40]. Mechanical stretch-induced skin regeneration, as observed during tissue expansion, relies on the ability of the epidermis and dermis to sense biomechanical cues and convert them into coordinated proliferative and remodeling responses [3]. In the epidermis, acute stretching directly stimulates nuclear β-catenin in affected KCs, enhancing their proliferation, thereby increasing the epidermal thickness and surface area to accommodate tissue expansion [36]. β-catenin also accumulates in the nuclei of the specialized dendritic LCs, which proliferate and migrate into the stretched suprabasal layer of the skin, rebuilding the growing epidermis. From there, LC secrete SFRP2 in order to restrain the enhanced canonical Wnt activation and stimulate KC proliferation, thus restoring the epidermal homeostasis disrupted by tissue expansion [41]. In the deeper dermal layer, Wnt signaling induces extracellular matrix remodeling, supporting the structural adaptation of stretched skin, by stimulating fibroblast to produce more collagen and increase their proliferation and migration (Figure 1A).

### 3.2. Wnt Signaling in Epidermal Response to Injury

Skin wound healing proceeds through three partially overlapping phases—inflammation, proliferation (re-epithelialization, tissue granulation, and angiogenesis), and tissue remodeling. Classic studies in the mice model system demonstrate that wounding induces local Wnt signaling, which then participates in these stages, regulating inflammation, controlling programmed cell death, and mobilizing multiple cell populations to undergo coordinated proliferation, differentiation, and migration to contribute to tissue repair, ultimately restoring the epidermal barrier, mechanical integrity, and overall tissue architecture [7]. Throughout this regenerative process, both complementary Wnt pathways are crucial—while the canonical Wnt signaling serves as a central coordinator of wound repair, supporting proliferative expansion and dermal granulation/EMC production, the non-canonical Wnt signals orchestrate epithelial polarity, directed migration, and inflammatory coupling [3,42].

#### 3.2.1. Wnt Signaling in Early Response to Injury—Inflammation and Proliferation

**Figure 3:**
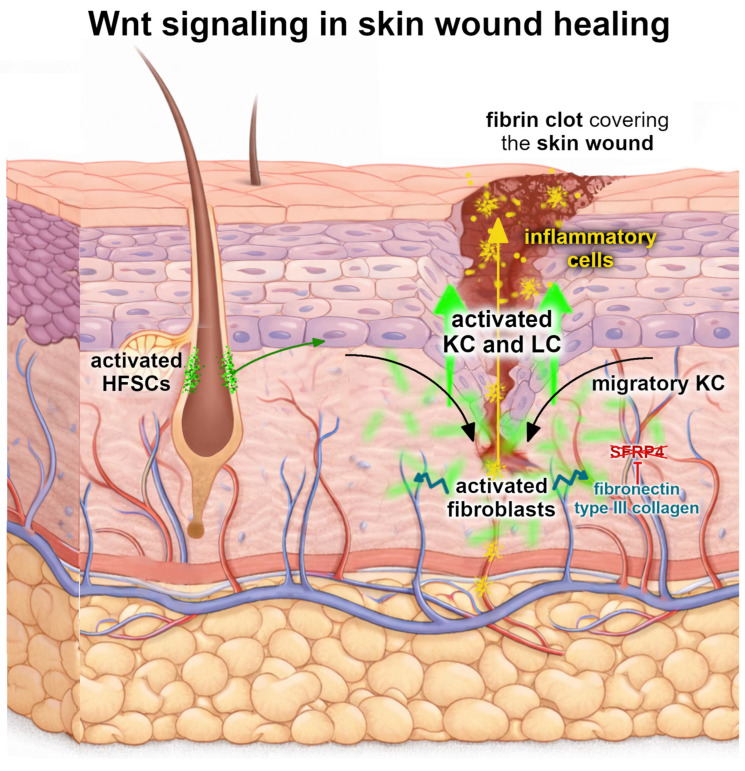
Activation of Wnt signaling pathway in wound healing. Following injury, Wnt activation induces KC, LC, and mesenchymal cells within the wound environment, stimulating their proliferation and migration. Through injured blood vessels, platelets and inflammatory cells gather at the fibrin clot from where their release of pro-inflammatory cytokines and growth factors is interrelated with increased Wnt signaling at the site (yellow arrow). The activation of fibroblasts in the deeper layer of skin facilitates their migration, proliferation, and secretion of growth factors and extracellular matrix components (EMC)—type III collagen and fibronectin. Fibronectin affects the SFRP4 Wnt inhibitor, promoting its degradation by macrophages, thus reducing local Wnt inhibition. Wnt signaling also affects the cell behavior of HFSCs, inducing their self-renewal and proliferation, so they can further differentiate into epidermal cells, thus promoting healing of the injured skin. KC—keratinocytes, LC—Langerhans cells, HFSCs—hair follicle stem cells, EMC—extracellular matrix components.

During normal skin homeostasis, the canonical Wnt pathway in KCs is vastly inhibited in the interfollicular epidermis, preventing their uncontrolled proliferation. In these conditions, stable β-catenin localized at the plasma membrane acts as a structural anchor for E-cadherin in adherens junctions, contributing to KCs’ adhesion connection and ensuring proper skin integrity. Upon injury, the Wnt pathway activates rapidly, most likely triggered by changes in the oxygen tension through hypoxia-inducible factor-1α (HIF-1α) [43,44], and is induced in KCs and mesenchymal cells within the wound microenvironment. This leads to the stabilization of β-catenin in the cytoplasm, allowing it to accumulate and translocate to their nucleus. There, β-catenin associates with TCF/LEF transcriptional complexes to regulate KC proliferation and differentiation [45,46,47,48]. This process also disrupts the intercellular connections between KCs by the degradation of E-cadherin on the cell surface, thereby facilitating cell motility (Figure 3). More recent mechanistic in vitro and mice in vivo studies also link canonical Wnt to epidermal adhesion structures—hemidesmosomes (HDs), whose timely assembly and disassembly is crucial during the re-epithelialization phase of wound healing [49]. An inhibition of canonical Wnt signaling was demonstrated to disturb HD organization in KC, thus suggesting that the Wnt pathway could become a potential therapeutic target for treating HD-defective skin disorders [49].

Skin injury is accompanied by bleeding which leads to the constriction of blood vessels and platelets gathering at the developing fibrin clot, where they release cytokines that induce the migration of the inflammatory cells to the site (Figure 3). Neutrophils, macrophages, and lymphocytes keep the wound free of bacteria, foreign bodies, and dead cells, but also release proinflammatory cytokines and growth factors that were observed to be linked to increased Wnt signaling [45]. However, rather than acting as a simple on/off switch for inflammation, Wnt signaling functions as a contextual regulator of immune responses during wound healing through crosstalk with NF-κB (Nuclear Factor kappa-light-chain-enhancer of activated B cells) and MAPK (mitogen-activated protein kinase) signaling. Early, localized activation, particularly through canonical Wnt ligands, supports the release of pro-inflammatory cytokines and growth factors necessary for effective skin repair [50], and, as healing progresses, the modulation of canonical Wnt activity contributes further to inflammation resolution and tissue remodeling. In the context of inflammatory cells participating in wound healing, the macrophages are the best-characterized immune cell type in which Wnt signaling has been functionally linked to cytokine and growth factor release. Both canonical Wnt and non-canonical Wnt pathways regulate macrophage activation states. In acute injury, non-canonical Wnt ligands such as WNT5A promote their pro-inflammatory phenotype, enhancing the production of cytokines including TNF-α, IL-6, and IL-1β, as well as growth factors such as VEGF and TGF-β, which are critical for angiogenesis and early tissue repair [45]. By contrast, canonical Wnt signaling has been shown to fine-tune the inflammatory output, limiting excessive inflammation and facilitating the transition toward tissue repair [28].

Finally, non-canonical Wnt signaling pathways, particularly the Wnt/PCP and Wnt/Ca^2+^ branches, are closely linked to processes such as to directed cell migration, tissue polarity, and inflammatory signaling, making them critical regulators of wound closure. The Wnt/PCP pathway coordinates the cell polarity and directs KC migration at the wound edge, thereby organizing a collective epithelial movement during re-epithelialization. Moreover, PCP signaling has been linked to mechanotransduction processes that translate mechanical cues into coordinated changes in cell polarity and cytoskeletal organization [51,52]. Similarly, crucial for KC migration in the wound microenvironment is the JNK signaling, which can be activated by non-canonical ligands and regulates the formation of lamellipodia (cellular projections) and the reorientation of the microtubule-organizing center (MTOC), both essential for polarized cell movement during wound repair as shown in both in vitro and in vivo studies [53,54,55]. This process involves cytoskeletal regulators such as actin-binding protein filamin A and PKC, which facilitate directional migration toward the wound site. The non-canonical ligand WNT5A, acting through receptors such as ROR2 and FZD, activates downstream signaling modules including the PCP pathway with RhoA, Rac1, ROCK, and JNK, as well as an additional polarity regulator such as Rap1, while parallel Ca^2+^-dependent signaling branches can engage PKC [53,56]. Accordingly, WNT5A is considered a major mediator of non-canonical Wnt signaling in wound repair, where it contributes to inflammatory regulation, promotes the migration of fibroblasts and epidermal stem cells, and can drive the fibroblast-to-myofibroblast transition associated with fibrotic remodeling [57].

Deeper in the skin, the wounded dermis is repaired by migrating and proliferating fibroblasts which also release growth factors and secrete extracellular matrix components such as type III collagen and fibronectin (Figure 3). Moreover, Wnt signaling intersects with other repair-associated pathways, including TGF-β and Notch, to modulate fibroblast activity and granulation tissue formation [3,24]. Studies on in vitro human cell lines and in vivo mice models using activators of the canonical Wnt pathway demonstrate enhanced wound healing outcomes, including increased KC and fibroblast migration, stem cell activation, and neo-epidermis formation, confirming a fundamental role for Wnt signaling in orchestrating regenerative processes [42].

#### 3.2.2. Wnt Signaling in Skin Remodeling

The remodeling phase is the final stage of wound healing, during which the provisional granulation tissue is progressively replaced by a more organized extracellular matrix (ECM), and tissue strength and function are restored. During this stage, which can last weeks to months after the initial injury, canonical Wnt signaling remains functionally relevant, contributing to the regulation of fibroblast activation, ECM deposition, and the resolution of inflammation. Moreover, fibronectin within the extracellular matrix can sequester the Wnt inhibitor SFRP4, promoting its uptake and degradation by macrophages. This process reduces local Wnt inhibition and thereby sustains Wnt signaling activity within the wound environment, corresponding with fibrotic wound repair [58].

However, the excessive stabilization of β-catenin has been reported to impair keratinocyte migration and delay wound healing [16]. Moreover, sustained canonical Wnt activity during the remodeling phase has been implicated in pathological scarring, keloid formation, and fibrosis. Consistent with this, human hypertrophic scars and keloids display elevated levels of β-catenin, and an increased expression of Wnt target genes in fibrotic skin, suggesting that prolonged Wnt signaling can skew tissue repair toward excessive extracellular matrix deposition and aberrant fibroblast activation [18]. Only appropriate downregulation supports scar maturation and the restoration of normal tissue mechanics [7,24]. Mechanistically, Wnt ligands and their receptors modulate key transcriptional programs in fibroblasts and immune cells that intersect with established remodeling pathways such as TGF-β and Hippo/YAP signaling [24,59]. For example, β-catenin can cooperate with TGF-β effectors to regulate the expression of collagen type I and III as well as matrix metalloproteinases, thereby influencing both matrix synthesis and breakdown [24]. At the stromal level, non-canonical Wnt signaling driven by WNT5A directly intersects with pro-fibrotic pathways, by promoting the activation of latent TGF-β. Mechanistic in vivo studies in mice and the analysis of human fibrotic tissues demonstrate that WNT5A signaling, through downstream effectors including JNK and ROCK, facilitates TGF-β activation, thereby driving fibroblast activation and extracellular matrix remodeling. This provides a mechanistic link by which sustained WNT5A activity may promote pathological fibrosis and scarring [57].

Importantly, the temporal regulation of Wnt activity is critical: while early wound healing is characterized by the transient activation of Wnt to support inflammation and re-epithelialization, the remodeling phase relies on precise temporal downregulation and the context-dependent modulation of Wnt activity to ensure a balanced matrix deposition and to avoid pathological scarring or fibrosis.

### 3.3. Dysregulation of Wnt Signaling in Skin Pathologies

Wnt signaling functions as a central coordinator of epidermal stem cell behavior and tissue dynamics under both physiological and pathological conditions. In disease, Wnt pathway activity is frequently mis-timed, mis-localized, or imbalanced, leading to pathological outcomes that range from chronic inflammation and defective regeneration to fibrosis and cancer. The collected evidence reveals that Wnt signaling disruption manifests differently across skin pathologies based on three key determinants: the specific pathway branch affected (canonical β-catenin-dependent versus non-canonical), the cellular context (epithelial cells versus stromal fibroblasts), and the presence of cooperating signaling pathways. Importantly, “Wnt dysregulation” in skin is rarely a single on/off event—rather, it reflects the context-dependent remodeling of ligand availability, receptor usage, extracellular antagonism, and downstream transcriptional outputs in both epithelial and mesenchymal compartments [3,24].

#### 3.3.1. Chronic Wounds, Impaired Repair, and Pathological Scarring

The prolonged stabilization of β-catenin has been reported to impair KC migration and delay wound healing, while also suppressing regenerative responses through the altered regulation of downstream target genes, including *c-Myc* [16]. One well-characterized pathology linked to dysregulated Wnt signaling is the development of chronic non-healing wounds, as observed in diabetic ulcers and aged skin. In these contexts, the normal transient activation of canonical Wnt signaling in early repair is disrupted, leading to insufficient proliferative and migratory responses from KCs and fibroblast [24]. Reduced or asynchronous Wnt signaling is associated with persistent inflammation, defective re-epithelialization, and an inability to progress to tissue remodeling, thereby predisposing it to ulceration and chronic wound states.

A related clinical problem is pathological scarring (hypertrophic scars and keloids), where fibroblasts and myofibroblasts sustain excessive ECM deposition. Recent work and reviews place Wnt signaling within a broader network that includes mechanotransduction (e.g., YAP/TAZ) and canonical fibrotic drivers (notably, TGF-β) [60]. In this framework, the Wnt output can promote fibroblast activation and matrix production, while cross-regulatory nodes can restrain Wnt to favor more regenerative, less fibrotic outcomes. This reinforces the idea that targeting Wnt in skin repair is feasible but must be precision-tuned to avoid pathological healing outcomes.

#### 3.3.2. Inflammatory Skin Diseases

An inflammatory skin disease, psoriasis, is characterized by KC hyperproliferation and immune infiltration, with strong cytokine-driven circuits. Multiple studies report the altered expression of Wnt components and antagonists in psoriatic lesions, and a commonly cited model proposes a shift away from canonical Wnt signaling toward non-canonical Wnt programs, accompanied by a reduced expression of certain canonical readouts/antagonists [3,61]. This shift is biologically plausible because non-canonical ligands, such as WNT5A, can remodel KCs’ behavior and inflammatory responses depending on the receptor context. Mechanistically, lesion-specific changes in Wnt antagonism (e.g., WIF1/DKK axis) and receptor availability are thought to reshape the pathway output in KCs and the dermal niche, with consequences for epidermal thickening and altered differentiation [61].

Another chronic inflammatory skin disorder, atopic dermatitis (AD) is characterized by recurrent eczematous lesions, intense pruritus, and impaired epidermal barrier function. The disease results from a complex interplay between genetic predisposition, immune dysregulation, and environmental factors, leading to abnormal KC differentiation and increased skin permeability. Experimental and translational studies suggest that, in at least some cases, β-catenin-dependent transcriptional activation may contribute to dysfunctionality in KCs, and that the pharmacologic modulation of β-catenin co-activator usage could alter AD-like inflammatory features in these models [62].

Genome-scale human studies increasingly implicate Wnt pathway components in disorders of follicular keratinization and chronic inflammation beyond classic inflammatory dermatoses. Recently, a genome-wide meta-analysis of hidradenitis suppurativa (HS)—a relapsing skin disease affecting intertriginous areas rich in hair follicles and apocrine glands—identified genetic loci associated with disease susceptibility. Several of these loci point to genes involved in Notch and canonical Wnt signaling pathways, supporting the concept that dysregulated epithelial differentiation and keratinization contribute to HS pathogenesis [63]. While HS is a multifactorial disorder that cannot be attributed to a single signaling pathway, these human genetic data provide additional support for the involvement of Wnt activity in disease biology and motivate mechanistic studies in epithelial models. Importantly, these observations are consistent with the emerging concept of Wnt–Notch crosstalk, sometimes referred to as “Wntch” signaling, which integrates multiple signaling inputs to regulate cell fate decisions in epithelial tissues [64].

#### 3.3.3. Pigmentary Pathology

Although vitiligo is classified as an autoimmune disease targeting melanocytes, repigmentation depends on the ability to regenerate melanocytes from precursor pools. Multiple sources highlight that canonical Wnt signaling supports melanocyte lineage regeneration, including the differentiation of melanocyte stem cells, and that lesional vitiligo skin shows evidence consistent with downregulated canonical Wnt activity [65,66]. This provides a mechanistic bridge between immune-mediated melanocyte loss and defective regenerative replacement, and supports therapeutic concepts that combine immune control with regeneration-promoting cues.

#### 3.3.4. Skin Cancers

In most skin cancers, canonical Wnt pathway activation drives tumor formation and growth; however, melanoma represents an exception where canonical Wnt activation paradoxically suppresses proliferation [67]. While nuclear β-catenin was observed to progressively decrease during melanoma progression, WNT5A was described to increase with the tumor grade, suggesting melanoma must suppress canonical Wnt signaling while activating non-canonical pathways to achieve maximal invasiveness [68].

Research focusing on non-melanoma skin cancers emphasizes that both canonical and non-canonical Wnt signaling can contribute to carcinogenesis, often through crosstalk with inflammatory and growth-factor pathways [19,69]. A particularly clear example are skin tumors with matrical differentiation, including pilomatricoma, where somatic activating mutations in *β-catenin* gene are frequent and drive hair matrix-like differentiation programs [15,61,70].

In basal cell carcinoma (BCC), constitutive canonical Wnt activation promotes tumor formation and growth, with canonical signaling required downstream of Hedgehog pathway activation [71]. In this established hierarchical relationship, Hedgehog provides the initial transforming event but requires Wnt pathway competence for tumor formation. This explains why *Dkk1* overexpression blocks BCC formation despite intact Hedgehog signaling. However, the composition and functional output of Wnt signaling vary across BCC subtypes. Superficial BCCs show enhanced canonical Wnt activity associated with hair follicle lineage features, whereas other subtypes display distinct alterations in Wnt ligands and signaling components. Both canonical and non-canonical Wnt pathways contribute to BCC pathogenesis, with non-canonical ligands such as WNT5A and WNT16B promoting tumor growth and invasion independently of β-catenin stabilization [72,73]. Moreover, the modulation of Wnt signaling outcomes by cofactors such as the vitamin D receptor influences the tumor phenotype, determining whether Wnt activation drives benign follicular tumors or malignant BCC [74].

Squamous cell carcinoma (SCC) is characterized by the constitutive activation of canonical Wnt/Tcf signaling, as evidenced by cytoplasmic and nuclear β-catenin accumulation and the upregulation of β-catenin target genes, including *c-Myc* and *c-Jun* [75]. Moreover, different tumor regions hold high levels of Wnt proteins including those encoded by *Wnt3*, *Wnt4*, and *Wnt10b*. The functional inhibition of this pathway suppresses SCC growth, indicating its essential role in tumor maintenance [75,76]. Beyond tumor cells, Wnt signaling within the tumor microenvironment critically contributes to SCC progression: Wnt-activated fibroblasts promote keratinocyte hyperproliferation and extracellular matrix remodeling through paracrine cytokine release and metalloproteinase induction [77]. In parallel, non-canonical WNT5A signaling drives SCC invasion and tumor maintenance through gradient-dependent migration and the activation of PKCα–STAT3 signaling, independent of β-catenin [73].

Another type of rare, malignant skin cancer arising from oil-producing glands called sebaceous carcinoma displays a distinct mode of Wnt pathway dysregulation driven by NH_2_-terminal *Lef1* mutations, which functionally inhibit canonical Wnt signaling [78]. These mutations accelerate tumor formation, bypass P53-dependent tumor suppression, and bias tumor differentiation toward sebaceous rather than squamous lineages. Together, these findings indicate that *Lef1* mutations act both as tumor promoters and lineage-specifying factors through the suppression of Wnt signaling [48].

A rare subset of cutaneous carcinomas characterized by the strong activation of the canonical Wnt signaling pathway without classic pilomatrical differentiation, such as Wnt/β-catenin-activated rosette-forming carcinoma, was recently described by Kervarrec et al. [79]. Their work highlighted that the distinctive histopathologic and molecular features, including nuclear β-catenin accumulation, suggest that aberrant Wnt activation can drive malignant epidermal tumors independently of hair matrix lineage commitment.

## 4. Wnt Signaling Dynamics in Hair Follicle Homeostasis and Hair Cycle

Canonical Wnt signaling is one of the central regulators of adult hair follicles (HFs) (Figure 1B). It is involved in stem cell homeostasis, maintenance, lineage commitment, and cyclical regeneration in both rodents and humans [80]. In adult mammalian skin, Wnt activity is finely regulated throughout the hair cycle. It remains low during the telogen phase and becomes selectively activated in the secondary hair germ (sHG) and hair matrix at the beginning of anagen [80,81]. This transition is characterized by the nuclear accumulation of β-catenin, which activates direct Wnt target genes such as *Axin2* and is coordinated with the activation and proliferation of hair follicle stem cells (HFSCs) [10,81,82]. Genetic and functional studies demonstrated that autocrine and paracrine epithelial Wnt ligands maintain HFSC proficiency, promote proliferation, and are crucial for anagen initiation. The loss of epithelial Wnt production or the attenuation of canonical Wnt signaling results in hair-cycle arrest and progressive hair loss [10,82].

Transgenic modification studies demonstrated canonical Wnt signaling as essential for postnatal hair regeneration. The conditional deletion of *β-catenin* in postnatal mice showed complete hair loss after the first hair cycle, as HFSCs fail to differentiate into follicular keratinocytes and instead adopt an interfollicular epidermal fate [83,84], whereas the inducible activation of stabilized ΔNβ-cateninER in the adult mouse epidermis under the keratin 14 promoter is sufficient to trigger rapid de novo hair follicle formation from both the interfollicular epidermis and existing follicles. This process necessitates downstream Sonic hedgehog signaling (Shh) [15,47,85]. These studies demonstrate that canonical Wnt signaling is both necessary and sufficient for hair follicle fate specification and regeneration.

Multiple Wnt ligands contribute to HF maintenance. Ligands, such as WNT10B, WNT7A/B, and WNT3A, play an important role in sustaining anagen and promoting HFSC activation [86,87,88,89,90]. Mouse studies demonstrated that *Wnt10b* overexpression in the skin robustly drives the telogen-to-anagen transition via canonical Wnt signaling. Temporal mapping further showed that the activity of *Wnt10b* first stimulates HFSC proliferation within 24 h, followed by the activation of the precortex, matrix compartments, and outer bulge cells by 72 h [91,92]. Contrariwise, the siRNA-mediated knockdown of Wnt10b or β-catenin prevents anagen entry, positioning *Wnt10b* as a key endogenous activator of hair follicle regeneration [91].

Moreover, Wnt signaling exhibits a high dosage sensitivity. The overexpression of *Wnt3* in the transgenic mouse skin induces a short-hair phenotype with cyclical balding by altering hair shaft precursor differentiation and causing structural defects. Dishevelled 2 (DVL2) mediates these effects within the outer root sheath of mouse hair follicles, demonstrating that excessive or mis-regulated Wnt signaling disrupts the hair shaft architecture rather than promoting regeneration [81,93]. Likewise, sustained *Wnt10b/β-catenin* overexpression in mice promotes hair follicle morphogenesis, pigmented regeneration, and wound-induced neogenesis; however, persistent and unrestrained pathway activation disrupts epithelial homeostasis, causing hyperproliferation and thus increasing the risk of skin tumorigenesis [94,95].

Physiological mechanisms can amplify Wnt signaling without increasing the oncogenic risk. In vivo studies in mice revealed that the intradermal administration of R-spondin-2, a potent Wnt agonist, enhances endogenous Wnt signaling and promotes hair regeneration, demonstrating that the controlled potentiation of Wnt signaling supports regeneration while avoiding pathological consequences associated with constitutive β-catenin activation [96].

Next, autocrine and paracrine Wnt signaling support the maintenance of the HFSC identity. Outer bulge HFSCs maintain a constant *Axin2* expression, reflecting a sustained autocrine Wnt activity that preserves the stem cell potency during quiescence and growth, whereas inner bulge enriched paracrine Wnt inhibitors, such as DKK3, promote differentiation and spatially restrict stemness [82].

The antagonistic BMP–Wnt interaction fine-tunes HFSC fate decisions. Inactivating BMP signaling in bulge HFSCs induces *Wnt7a/b*, *Wnt16*, and *Fzd10* expression, revealing an intra-stem cell BMP–Wnt antagonism that shifts cells along a continuous BMP/Wnt ratio gradient towards a hair germ fate [90]. At the transcriptional level, TCF3/4-TLE complexes repress Wnt target genes to maintain HFSC quiescence and stemness, whereas canonical Wnt activation lifts this repression and promotes proliferation, hair follicle fate commitment, and the suppression of sebaceous differentiation [97]. BMP inhibition combined with Wnt signaling establishes permissive chromatin landscapes, enables TCF3/4 replacement by LEF1 at multipotent enhancer loci, activates hair follicle progenitors, and enforces lineage specificity [98]. Hair germ cells derived from bulge HFSCs exhibit a precocious Wnt responsiveness and respond rapidly to dermal papilla (DP)-derived fibroblast growth factors (FGFs) and BMP inhibitors to initiate hair regeneration, while bulge HFSCs sustain long-term anagen progression [99]. Importantly, Wnt signaling functions in both epithelial and mesenchymal compartments: DP cells require intact β-catenin signaling to maintain their inductive capacity, while epithelial Wnt signaling orchestrates HFSC activation and lineage commitment. This bidirectional epithelial–mesenchymal Wnt crosstalk ensures the robust and coordinated regeneration during each hair cycle [11,100].

In addition to canonical signaling, non-canonical Wnt pathways modulate hair cycling. Bulge and hair germ cells express *Wnt5a*, which antagonizes canonical Wnt signaling, prolongs the telogen phase, maintains quiescence, and still allows eventual normal hair regrowth [101]. Furthermore, non-canonical Wnt signaling via ROR2-PKC activates and self-renews HFSCs, preserving the stem cell pool, when β-catenin is not present [102].

Negative feedback regulators of Wnt signaling maintain system stability. The loss of *Sfrp1* increases β-catenin activity, accelerates early hair cycles, and transiently depletes HFSCs, whereas subsequent BMP-AKT-GSK3β-mediated β-catenin downregulation, via its degradation, restores the stem cell pool, establishing *Sfrp1* as a key modulator of HFSC homeostasis in mice [103]. Similarly, CXXC5 binds Disheveled to suppress canonical Wnt signaling; the genetic deletion or peptide-mediated disruption of this interaction enhances Wnt activity, accelerates hair regrowth, and promotes wound-induced follicle neogenesis, identifying this axis as a therapeutic target for hair loss [104].

Canonical Wnt signaling also coordinates pigmented hair regeneration. Intrinsic Wnt activation drives melanocyte stem cell (McSC) proliferation and differentiation, while epithelial stem cell secretes Wnt ligands that couple melanocyte activation to epithelial hair growth through EDN–EDNRB (Endothelin–Endothelin receptor type B) signaling, synchronizing regeneration within the surrounding niche [105]. Selective *β-catenin* activation in HFSCs can even bypass classical DP dependence, inducing non-cell-autonomous Wnt ligand secretion that recruits neighboring wild-type epithelia into a coordinated regenerative program [106].

Human studies reveal the strong conservation of the Wnt pathway with species-specific features. During the telogen-to-anagen transition in human scalp follicles, Wnt signaling is activated via the upregulation of *WNT3*, *WNT4*, *WNT10B*, *WLS*, *AXIN2*, and *LEF1* in the secondary hair germ and dermal papilla, the downregulation of inhibitor *SFRP1*, and an increased *WNT4* and *WNT10A* expression in epithelial stem cells [87]. This human-specific Wnt switch provides a molecular rationale for Wnt-targeted hair growth therapies. Furthermore, canonical WNT3A/β-catenin signaling in human DP cells induces prostaglandin receptor *EP2* expression, enhances cAMP responsiveness, and supports anagen maintenance [107]. In human outer root sheath cells, constitutive *β-catenin* activation induces *KRT16* and *KRT17* expression via *PITX2*, establishing *PITX2* as a key downstream effector of Wnt-driven follicular differentiation [108]. Overall, it establishes WNT3A/β-catenin signaling as a central regulator of human follicular growth and lineage specification.

Wnt signaling can be modulated therapeutically in pathological contexts. Retinoic acid (RA) deficiency impairs HFSC function and hair regeneration in androgenetic alopecia (AGA), whereas RA treatment reactivates quiescent HFSCs through canonical Wnt signaling, and promotes anagen entry, highlighting the importance of early intervention [109]. Similarly, human-umbilical-cord-derived mesenchymal stem cells (hUCMSCs) promote AGA hair regeneration by secreting Wls-dependent Wnt ligands that activate canonical Wnt signaling in dermal papilla cells, reduce apoptosis, enhance growth factor production, and relieve the DP-mediated inhibition of HFSC proliferation. Their Wnt ligands also counteract DHT-induced DP apoptosis and improve the HFSC niche through paracrine mechanisms [110].

### 4.1. Biomechanical Control of Wnt Signaling in Hair Follicle Regeneration

The accumulated evidence suggests that hair follicles reside within a dynamic mechanical niche where mechanotransduction actively regulates canonical Wnt signaling during hair regeneration. Dermal stiffness, extracellular matrix (ECM) remodeling, arrector pili muscle contraction, and wound-induced tension generate mechanical cues that regulate HFSC behavior by converging on canonical Wnt signaling pathways [8,111,112]. Alterations in the tissue tension can directly stabilize β-catenin, thereby lowering the activation threshold for Wnt signaling and promoting HFSC activation and entry into anagens [113].

At the cellular level, integrin-mediated signaling, focal adhesion dynamics, and cytoskeletal tension regulate Wnt responsiveness in HFSCs, while ECM and basement membrane remodeling control the Wnt ligand availability and distribution to fine-tune pathway activation [114,115]. Together, these support a model in which Wnt signaling integrates biochemical and biomechanical cues to adapt the hair follicle regeneration output to the local mechanical environment [8].

In wound-induced hair neogenesis (WIHN), Wnt signaling integrates morphogenic and mechanoregulatory functions. The amplified canonical Wnt activity in the wound epidermis remodels chromatin, softens keratinocyte nuclei, reorganizes the actin-adherens junction into a cohesive mechanical syncytium, enhances force transmission and durotaxis, optimizes tissue rigidity, and promotes regenerative HF formation over fibrotic scarring [8].

The manipulation of tissue mechanics further demonstrates the importance of mechanotransduction in Wnt-driven regeneration. Reducing mechanical tension enhances de novo follicle formation in WIHN models, as softer central wound regions or focal adhesion kinase (FAK) or myosin II inhibition decrease tissue stiffness, downregulate cytoskeletal-integrin-matrix programs, increase nuclear pSTAT3 signaling, and expand the HF neogenesis [116]. In contrast, the reinforcement of the actin-mediated tension suppresses regeneration, underscoring that mechanical permissiveness is required for effective Wnt-mediated follicle formation [8].

Mechanical stimulation can also promote hair regeneration within an optimal biomechanical window. Controlled skin stretching in mice stimulates HFSC proliferation by rebalancing Wnt and BMP2 signaling, recruiting M2 polarized macrophages, and inducing the release of growth factors, such as HGF and IGF-1. These activate HFSCs, and establish mechano-immune-Wnt cascades that stimulate follicle regrowth [111]. Similarly, mesenchymal stem cell (MSC)-based therapies, MSC secretomes, and platelet-rich plasma leverage dermal papilla–mesenchymal interactions promote follicle regeneration, chimeric follicle formation, and neogenesis [112].

Species-specific differences in WIHN further highlight the role of tissue mechanics in shaping Wnt-driven regeneration. Unlike *Mus musculus*, *Acomys* create larger, stiffness-controlled healing areas. In these areas, Twist1-driven communication between the epidermal–dermal areas, together with a two-step Turing-like chemo-mechanical process, allows more new hair follicles to form. This finding is important for regenerative medicine [117,118].

Lastly, emerging evidence shows that bioelectric–mechanical coupling adds an extra regulatory layer to Wnt responsiveness during hair regeneration. Chromatin rearrangements that upregulate the potassium channel KCNJ2 in dermal fibroblasts induce membrane hyperpolarization, reduce intracellular calcium levels, and increase Wnt signaling sensitivity. This bioelectric state is naturally found during the anagen phase, and experimentally driving KCNJ2-dependent hyperpolarization rescues hair loss in aging and androgenetic alopecia models, establishing fibroblast bioelectricity as a novel and druggable regulator of Wnt-mediated hair regeneration [119].

### 4.2. Crosstalk Between Wnt and Regulatory Pathways in Hair Follicle Cycling

Hair follicle regeneration is governed by a highly integrated signaling network in which canonical Wnt signaling functions as a central regulatory hub rather than a linear pathway [10,89]. Multiple signaling cascades act both upstream and downstream of Wnt to modulate stem-cell quiescence, activation, lineage commitment, and tissue remodeling [80,95,120,121].

**Figure 4:**
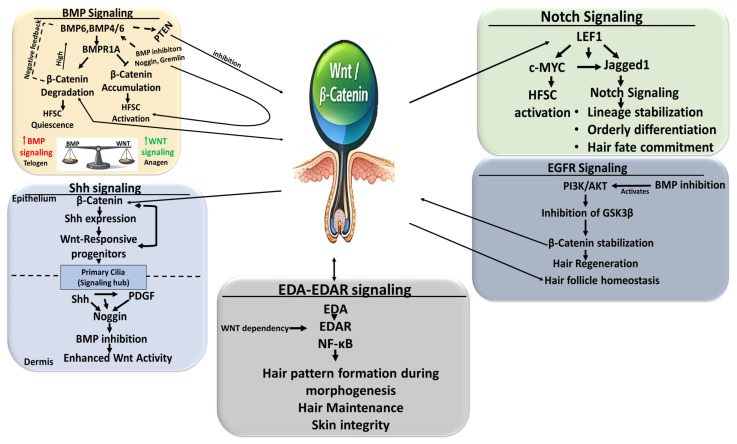
Crosstalk of key signaling pathways with Wnt signaling, resulting in regulation of hair follicle behavior and hair cycle. BMP signaling maintains HFSC quiescence through β-catenin degradation, while BMP inhibition leads to β-catenin accumulation and HFSC activation. Wnt and Shh signaling promote progenitor activation and hair growth, with primary cilia acting as signaling hubs. Notch signaling involving LEF1, c-MYC, and Jagged1 supports HFSC activation, lineage stabilization, and differentiation. PI3K/AKT/and EGFR pathways inhibit GSK3β, stabilizing β-catenin and maintaining hair follicle homeostasis. WNT-dependent EDA–EDAR signaling is essential for maintenance, hair pattern formation, and skin integrity.

BMP (Bone Morphogenetic Protein) signaling is the dominant negative regulator of Wnt activity in the HFSC niche. During the telogen phase, elevated BMP signaling maintains HFSC quiescence by suppressing β-catenin-dependent transcription, while localized BMP inhibition (e.g., Noggin and Gremlin) permits Wnt activation and anagen entry. The BMP6–WNT10B balance quantitatively determines the telogen–anagen transition rather than an on/off switch [90,122]. The loss of *Bmpr1a* in mice HFSCs leads to nuclear β-catenin accumulation and precocious HFSC activation, demonstrating that BMP gates Wnt competence upstream [121]. Conversely, the excessive canonical Wnt activation in dermal stem cells induces *Bmp4/6*, establishing BMP as a downstream feedback regulator. BMP signaling also restrains epithelial stem-cell activation via PTEN-mediated Wnt antagonism, while BMP inhibition triggers pathological HFSC expansion (Figure 4) [90,120,121].

Canonical Wnt signaling initiates HFSC activation and hair germ formation, while Shh (Sonic hedgehog) signaling sustains the proliferation of transit-amplifying and matrix cells during anagen [95]. Wnt induces *Shh* expression, and Shh amplifies Wnt-responsive progenitors, forming a regenerative feedback loop. Primary cilia coordinate this interaction, as Shh and PDGF signaling promote *Noggin* expression and indirectly enhance epithelial Wnt activity (Figure 4) [95,123].

Notch signaling cooperates with Wnt to stabilize the lineage commitment and prevent premature differentiation. Wnt activates HFSCs, while Notch ensures orderly differentiation and follicular integrity. Lef1-dependent Wnt signaling activates c-Myc and Jagged1/Notch signaling, promoting hair fate, whereas, in the case of the β-catenin-binding-deficient Lef1 mutant, such an effect does not occur in mice [95,124]. Notch inhibition reduces Wnt-induced ectopic follicle formation, indicating that Notch acts permissively downstream of Wnt signaling (Figure 4) [95].

EGFR (Epidermal Growth Factor Receptor) signaling functions as an upstream brake on Wnt; EGFR loss causes excessive *β-catenin* activation and follicular degeneration, whereas restoring Wnt antagonists (e.g., SFRP1) rescues these defects in mice. In androgenetic alopecia, DHT suppresses Wnt and activates TGF-β/Smad signaling, and reciprocal inhibition rebalances these pathways, highlighting the therapeutic potential [125,126]. Multiple pathways converge on Wnt via the PI3K-AKT-mediated inhibition of GSK3β, stabilizing β-catenin and integrating the growth factor with BMP and mechanical inputs (Figure 4) [103,121].

In adult tissue, the canonical Wnt pathway induces the action of ectodysplasin A (EDA) and its receptor (EDAR), which maintains skin appendages, homeostasis, and regeneration, while its disruption compromises hair cycling and epithelial integrity (Figure 4) [127].

Bi-directional epithelial–mesenchymal signaling coordinates the Wnt output. Wnt ligands preserve the dermal papilla inductive capacity, whereas excessive dermal *β-catenin* activation reprograms fibroblasts into fibrogenic states, induces BMP, FGF, and Notch ligands, and promotes ectopic follicles and fibrosis [80,95].

### 4.3. Pathological Consequences of Wnt Signaling Dysregulation in Hair Follicle

Given its central role in HF maintenance and regeneration, the tight spatial and temporal regulation of canonical Wnt signaling is essential. Both the hypo- and hyperactivation of this pathway result in pathological outcomes (Table 1). Insufficient Wnt activity impairs HFSC activation, delays anagen entry, and promotes follicle miniaturization characteristic of alopecia. Attenuated β-catenin signaling compromises matrix cell proliferation and reduces the DP inductive capacity, resulting in shortened hair cycles, decreased hair shaft production, and progressive hair thinning [10,128,129].

Among hair disorders, androgenetic alopecia (AGA) is the most extensively characterized condition in which impaired Wnt signaling drives follicular degeneration [25]. Multiple studies demonstrated that reduced canonical Wnt activity shortens anagen duration, suppresses HFSC proliferation, and accelerates the premature transition into catagens and telogens [84,88,121,130,131]. In AGA, androgen signaling directly antagonizes canonical Wnt activity in the DP. The dihydrotestosterone (DHT)-activated androgen receptor physically interacts with β-catenin, diverts it from TCF/LEF-mediated transcription, and induces Wnt antagonists such as *DKK1*. Elevated *DKK1* in the balding scalp correlates with reduced anagen follicles and increased keratinocyte apoptosis, establishing androgen-mediated Wnt repression as a central pathogenic mechanism [110]. Furthermore, transcriptomic analysis reveals the global downregulation of Wnt target genes, including *AXIN1* and *LEF1*, indicating that reduced Wnt ligand responsiveness, rather than the loss of β-catenin itself, drives follicular regression [89,132].

Beyond androgen interference, oxidative stress and mitochondrial dysfunction further exacerbate Wnt pathway suppression in hair pathologies. The elevated reactive oxygen species (ROS) in DP cells from balding follicles destabilize β-catenin and reduce transcriptional activity, thereby compromising HFSC self-renewal and the regenerative capacity. Persistent oxidative stress disrupts the protective crosstalk between Wnt, Nrf2, and autophagy pathways, leading to premature HFSC senescence, shortened anagen, and follicular dormancy. Concurrently, mitochondrial dysfunction perturbs ROS-sensitive regulators such as GSK-3β activity and β-catenin degradation machinery, establishing a feed forward loop that accelerates hair loss progression [133].

Conversely, chronic or excessive Wnt activation is equally detrimental, driving aberrant follicular differentiation and tumorigenesis [10,130]. Sustained β-catenin signaling in epidermal or follicular compartments induces ectopic hair-follicle-like structures, sebaceous gland hyperplasia, and cystic or comedo-like lesions. Mouse models with constitutive Wnt activation develop follicular cysts and tumors, highlighting the oncogenic potential of unrestrained Wnt signaling in skin [15,88,130]. These findings underscore that tightly balanced Wnt activity is essential for preserving follicular architecture and lineage fidelity.

Wnt dysregulation also contributes indirectly to pigmentary disorders and hair graying by disrupting the coordination between epithelial HFSCs and melanocyte stem cells (McSCs). In mice, the loss of epithelial Wnt ligand production uncouples epithelial regeneration from melanocyte renewal, resulting in depigmented and structurally abnormal hairs. This highlights the broader role of Wnt signaling in synchronizing multiple stem cell populations within the hair follicle bulge [105].

Importantly, the dysregulated Wnt signaling in hair pathologies is not confined to epithelial compartments but reflects defective epithelial–mesenchymal communication. Dermal papilla cells require intact Wnt responsiveness to maintain their inductive function, and reduced epithelial Wnt ligand secretion weakens DP signaling and follicle regeneration. Conversely, fibroblast-derived non-canonical Wnt ligands (e.g., WNT5A) antagonize β-catenin signaling, prolong HF quiescence, and contribute to delayed or incomplete regeneration in pathological states [81,94,131].

Collectively, these findings establish dysregulated Wnt signaling as a central molecular hallmark of hair follicle disorders, integrating hormonal, metabolic, inflammatory, and oxidative stress cues within the follicular niche. Accordingly, therapeutic strategies aimed at restoring physiological Wnt signaling dynamics by relieving androgen mediated repression, reducing oxidative and mitochondrial stress, and modulating upstream regulators to re-establish a permissive regenerative environment in AGA and related pathologies [25,109,133].

**Table 1:** Genetic and pathological models of Wnt signaling in skin and hair. This table summarizes key genetic conditions, experimental models, and syndromes related to WNT signaling in skin and hair follicles. It covers both human and mouse studies, highlighting how mutations or manipulations in key Wnt signaling pathway genes lead to a range of phenotypes.

Condition/Model	Species	Manipulated Gene	Phenotype	Key Refs.
Odonto onycho dermal dysplasia	Human	*WNT10A*	Tooth agenesis, benign skin tumor, affecting hair	[134]
Schoof–Schulz–Passarge syndrome/related *WNT10A* ectodermal dysplasia spectrum	Human	*WNT10*	Palmoplanter keratoderma, nail, hair, teeth abnormalities	[135]
Conditional knockout of *Porcn* (related to Goltz syndrome)	Mouse	*Porcn* (required for Wnt screction)	Mutation causes spectrum of limb, skin, and body pattern abnormalities in females	[136]
Focal dermal hypoplasia (Goltz syndrome	Human	*PORCN* (Wnt ligand acyltransferase required for Wnt secretion)	Developmental skin defects, hairlessness	[137]
Hereditary hypotrichosis simplex (HHS), generalized form	Human	*APCDD1* (membrane Wnt inhibitor	Progressive hair follicle miniaturization—sparse hair/hair loss	[138]
*AXIN2*-associated ectodermal dysplasia/neoplastic syndrome	Human	*AXIN2*	Multisystem ectodermal phenotype—abnormalities in hair skin, nail, and tooth	[139]
Pilomatricoma	Human	*CTNNB1* (*β-catenin*)	Benign skin tumor, hair matrix-like differentiation	[140]
Truncated *β-catenin* in skin	Mouse	*Ctnnb1* (*β-catenin*)	De novo hair follicle morphogenesis and hair follicle tumors	[15]
Transgenic mice model expressing activated *β-catenin* (Gain of function)	Mouse	*Ctnnb1* (*β-catenin*)	Skin tumors resembling pilomatricomas	[20]
Transient *β-catenin* activation in adult epidermis (inducible system)	Mouse	*Ctnnb1* (*β-catenin*)	Induced new hair follicles	[47,85]
Epidermal Wnt secretion defect (*Wntless/Gpr177* deletion) (conditional loss of function)	Mouse	*Gpr177*/*Wls* (*Wntless*)	Affected hair morphogenesis	[141]
Conditional *β-catenin* knockout in epidermis	Mouse	*Ctnnb1* (*β-catenin*)	Hair loss after 1st cycle after deletion in epidermis	[84]
Apc (adenomatous polyposis coli) loss of function in epidermis	Mouse	*Apc*	Abrerrant follicle growth, hair follicle defects, ectopic epithelial phenotypes, tumor-prone contexts	[142]
Homozygous germline *Lef1* mutation	Mouse	*Lef1*	Mutate mice lack teeth, mammary gland, whiskers, and hair	[143]
*Fzd6* (*Frizzled-6*) knockout	Mouse	*Fzd6*	Hair follicle orientation/coat patterning	[144]
*Rspo3* overexpression	Mouse	*Rspo3*	Sparse hair and visible baldness	[145]
Conditional knockout of *Wnt7b*	Mouse	*Wnt7b*	Shorter anagen, premature catagen, and delayed hair follicle activation, affecting both hair germ bulge HFSCs	[86]
Overexpression of *Wnt7a*	Mouse	*Wnt7a*	Increased numbers of hair follicles	[146]

## 5. Wnt Signaling in Nail Mini-Organ Stem Cells and Digit Tip Regeneration

The nail mini-organ is a highly specialized ectodermal structure composed of the nail proximal fold (NPF), nail matrix, nail bed, and nail plate (NP), supported and sealed by the eponychium, hyponychium, and surrounding perionychium (Figure 1C). Together with the underlying distal phalanx (P3) bone, periosteum, nerves, and vasculature, these structures form a tightly integrated regenerative unit at the distal tip of each digit [13]. The continuous growth of each nail unit depends on the tightly regulated proliferation and lineage progression within the nail matrix epithelium. Its proximal area harbors a population of highly proliferative nail stem cells (NSCs) that differ molecularly from the interfollicular epidermal and HFSCs, but share a strong dependency on canonical Wnt signaling, which governs their differentiation and ensures continuous NP production.

### 5.1. Wnt-Dependent Control of Nail Growth and Differentiation

Wnt signaling exhibits distinct spatial organization within nail mini-organs. Highly proliferative Lgr6-positive NSCs are confined to the proximal region of the nail matrix where they express nuclear β-catenin in their nucleus and cytoplasm, in contrast to other adjacent epithelial cell types in which β-catenin shows only membrane localization [147]. However, the Wnt pathway activity—reflected by the presence of TCF/LEF transcription factors, Wntless and Wnt-responsive keratins—is spatially restricted to the distal matrix (Figure 1C) [12]. Positioned beneath both the nail matrix and the nail bed, in close proximity to Lgr6+ nail matrix cells, lies the onychodermis, rich in Rspo4-positive onychofibroblasts. Given that *Lgr6* is a Wnt-responsive stem cell marker that further enhances Wnt signaling via R-spondin-Lgr interactions, the communications between these compartments provides critical signaling cues, essential for nail formation during homeostatic growth [148,149,150]. This is additionally evidenced by the RSPO4 treatment of human nail matrix KCs, which resulted in the upregulation of *LGR6* and *FOXQ1*—markers of Wnt signaling activation in vitro [150].

Towards the tip of the digit, the nail matrix smoothly transitions into the nail bed, which was recently discovered to likewise differentiate from the nail matrix stem cells due to the expression of *Sox9* transcription factor [151]. However, increased levels of *WNT5A* and *BAMBI*—known antagonists of the canonical Wnt and BMP pathways, respectively—observed within the human nail bed onychodermis, suggests yet another regional modulation of Wnt and BMP signaling intensity in the nail unit [152].

Collectively, these observations led to the conclusion that the distal matrix serves as the primary Wnt-activation zone, which—supported by signals from the underlying nail bed—sustains the continuous differentiation of NSCs into NP onychocytes (terminally differentiated KC of the NP) and coordinates mesenchymal remodeling in the digit tip (Figure 1C) [12]. Indeed, loss-of-function mouse models have demonstrated that the genetic ablation of *β-catenin* in Krt14+ nail epithelia or disrupted Wnt ligand secretion (via Wntless) leads to severe nail growth pathologies. In the β-catenin-deficient epithelium, NP formation fails, leading to the expansion of undifferentiated, proliferative matrix cells, the loss of keratinization markers such as AE13, and structural defects of the distal phalanx [12,153].

The following studies identified an additional slow-cycling stem cells population within the nail proximal fold (NPF) epithelium that contributes long-term to the renewal of the overlying peri-nail epidermis and, upon injury, can actively participate in the regeneration of the damaged nail matrix and reconstruction of the NP [13,154]. Adjacent to these NPF stem cells (NPFSCs), and across the distal groove, a unique mesenchymal population expressing *Lgr5* has been identified [149,154]. *Lgr5* is a canonical Wnt target gene and an R-spondin receptor that can potentiate the Wnt signaling output, consistent with its broader association with highly Wnt-responsive stem cell populations. However, while epithelial Wnt activation is clearly required for nail growth and regeneration, the extent to which Wnt signaling directly governs NPFSC activation—and whether the neighboring Lgr5-positive mesenchymal cells constitute a functional niche component controlling nail growth and homeostasis—remains unresolved [12].

### 5.2. Role of Wnt Signaling in Digit Tip Regeneration

The mouse digit tip is an established model of epimorphic regeneration, and its ability to regenerate bone, nail, and connective tissues after shallow amputation depends critically on intact epithelial canonical Wnt signaling [12,13]. Distal amputations preserve both the NSC population and the adjacent Wnt-active domain in the distal Mx, which, together, define the permissive zone for regeneration, whereas proximal amputations remove the Wntless-expressing distal matrix and periosteal Wnt-responsive mesenchyme, rendering the stump regeneration-incompetent even when NSCs remain [13,153,155]. Forced β-catenin stabilization in the proximal epithelium rescues the regeneration only when applied after wound re-epithelialization and within the distal matrix region, where competent Wnt-responsive epithelial and mesenchymal cells are localized. When *β-catenin* activation is induced proximally, outside this permissive zone, neither osteoblast proliferation nor bone regrowth is restored, underscoring the strict spatial boundaries of Wnt responsiveness during regeneration [12,155].

**Figure 5:**
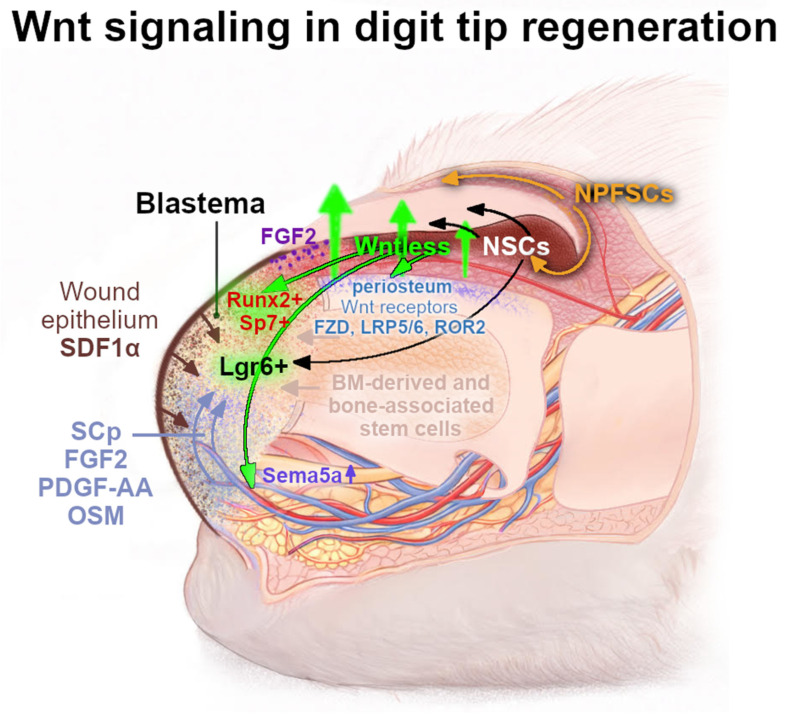
Wnt signaling in regenerating digit tip following digit injury. Digit injury that leaves a proximal matrix with NSCs and distal matrix with the intact Wntless activation zone enables NSCs to continue proliferating and differentiating into the NP. Moreover, NPFSCs also start actively participating in this process, replenishing the proximal matrix. The expression of Wntless protein by a distal nail matrix activates the Wnt signaling pathway, which promotes blastema growth—a structure formed by undifferentiated and highly proliferative cells including Lgr6-positive cells. Blastema is outlined by wound epithelium, which, through secretion of SDF-1, acts as signaling center for blastema cells. Wntless protein also promotes blastema innervation through upregulation of axon-guidance genes, such as *Sema5a*, thus enabling nerves and Schwann cell precursors (SCps) to reach the injured site and express nerve-derived factors such as FGF2, PDGF-AA, and OSM. Within 3 weeks post injury, the FGF2 is strongly induced in the distal area of regenerating nail epithelium (purple). Wnt ligands from nail matrix furthermore support regeneration of the P3 bone by engaging periosteal Wnt-responsive mesenchymal cells, crucial for distal appositional bone growth and extension of the nail bed. β-catenin stabilization additionally activates the proliferation of Runx2+ progenitors and Sp7+ osteoblasts responsible for bone formation and regeneration. NSCs—nail stem cells; NPFSCs—nail proximal fold stem cells; SDF1α—Stromal Cell-Derived Factor-1α, also known as CXCL12; SCps—Schwann cell precursors; FGF2—Fibroblast Growth Factor 2; PDGF-AA—Platelet-Derived Growth Factor AA; OSM—Oncostatin M; *Sema5a*—Semaphorin 5A.

Wnt activation in nail progenitors was described to be crucial both for nail regeneration and for attracting nerves that promote mesenchymal blastema growth, which highlights a dual role for Wnt signaling in coordinating both epithelial (nail) and mesenchymal (digit) tissue regeneration (Figure 5) [12]. The formation of blastema—an accumulation of a mass of undifferentiated, highly proliferative cells acting as a regenerative “sprout”—is a crucial step in the regeneration of a lost digit tip [13,156,157]. The Lgr6-expressing stem cells were identified to accumulate within this structure and contribute to the reformation of the bone, nail epithelium, and glandular structures. Lgr6-deficient mice display partial regeneration defects, including a disorganized nail epithelium and reduced bone regrowth, supporting a requirement for Lgr6-dependent Wnt responsiveness during regeneration [149].

Wnt signaling activation was also determined to be crucial in promoting blastema innervation through the expression of the Wntless protein by the distal matrix. A properly innervated injury site provides a migration route for the Schwann cells precursors (SCps) and secretes growth factors that stimulate blastema cells to proliferate and differentiate (Figure 5) [12]. Moreover, epithelium-derived Wnt ligands from the nail distal matrix are required for the maintenance and regeneration of the underlying digit bone by engaging periosteal Wnt-responsive mesenchymal cells, which are crucial for the distal appositional bone growth and extension of the nail bed [155,156]. Indeed, in β-catenin-deficient epithelial cells in the mice digit, the regeneration, even following a shallow amputation, is completely blocked—bone fails to regrow, the proliferation of Runx2+ progenitors and Sp7+ osteoblasts is reduced, FGF signaling is suppressed, and regenerating nerves fail to reach the injury site [12,149,155]. Defective innervation reflects the insufficient Wnt-dependent induction of axon-guidance genes such as *Sema5a*, which is normally upregulated through Wnt signaling during early regeneration, which results in FGF2 expression, especially strongly induced in the distal area of the regeneration nail epithelium by the third week following amputation (Figure 5) [12]. The subsequent loss of nerve-derived factors including FGF2, PDGF-Aa, and OSM impairs blastema proliferation and mesenchymal remodeling [158]. As a consequence, blastema formation collapses, and bone regrowth is abrogated. Conversely, the stabilization of β-catenin after wound closure was observed to enhance *TCF1* expression, restore innervation, increase FGF2 production, and reactivate the proliferation of Sp7+ and Runx2+ mesenchymal cells, leading to complete bone regeneration in distal amputations in mice [12].

The most recent disease-model work further proved the essential role of Wnt activity in NSC differentiation and digit regeneration, additionally revealing a therapeutic potential of Wnt modulation [159]. In a study performed by Cui et al., a virus-induced impairment of Wnt signaling was identified to result in halted nail regeneration, while the pharmacologic activation using GSK3β inhibitors such as CHIR99021 restored stem-cell differentiation and promoted the recovery of the NP in mice [159].

### 5.3. Dysregulation of Wnt Signaling in Nail Pathologies

In humans and mice, impaired Wnt signaling is consistently associated with congenital and syndromic nail abnormalities, reinforcing the requirement for canonical Wnt signaling in nail organ morphogenesis and maintenance. The loss of function of the Wnt receptor *Fzd6* was observed to disrupt nail differentiation and led to characteristic defects in nail morphology, accompanied by the flawed expression of keratins, increased Wnt antagonists such as DKK4, and altered Hedgehog/BMP pathway activity in mice and humans [160,161]. Furthermore, mutations in *Rspo4*, which encodes the Wnt-potentiating R-spondin 4, was identified to cause anonychia or hyponychia, highlighting the requirement for the R-spondin-mediated enhancement of Wnt signaling during embryonic nail development and postnatal homeostasis in mice [162,163]. The biallelic loss-of-function mutation in *WNT10A*, a key epithelial Wnt ligand, was reported to cause odonto-onycho-dermal dysplasia [164] and relate to ectodermal dysplasia phenotypes in which nail hypoplasia, dystrophy, or fragility are recurrent features, often accompanying defects in teeth, hair, and sweat glands [127]. A functional analysis of *WNT10A* variants demonstrated reduced β-catenin-dependent transcriptional activity and the impaired proliferation of epithelial progenitors, consistent with insufficient Wnt signaling in ectodermal appendages, including nail units in mice and humans [164]. The disruption of Wnt ligand secretion due to pathogenic variants in *PORCN* was observed in focal dermal hypoplasia (Goltz syndrome), where structural nail defects frequently occur as part of a broader appendage phenotype [136,137]. Moreover, biallelic variants in *KREMEN1*, a co-receptor for the Wnt antagonist DKK1, have been linked to ectodermal dysplasia with variable nail involvement, supporting the concept that not only ligand availability but also the fine-tuned regulation of canonical Wnt signaling intensity is critical for normal nail development [165]. Collectively, these human genetic disorders provide converging evidence that both a reduced Wnt signal output and impaired pathway modulation translate into clinically recognizable nail phenotypes, supporting conclusions drawn from experimental animal models. A comprehensive overview of genetic, infectious, and pharmacological perturbations of Wnt signaling affecting nail homeostasis, and regeneration in murine experimental models and humans is summarized in Table 2.

The recent molecular profiling of onychomatricoma (OM) using digital spatial transcriptomics and single-cell RNA sequencing demonstrated that OM is a nail unit tumor predominantly composed of onychofibroblasts, a specialized nail-associated mesenchymal cell population [166]. Spatial analysis showed that the stromal compartment of OM exhibits a significant upregulation of genes associated with Wnt pathway activation. *LGR6* expression was enriched in the basal layer of the overlying epithelial component, whereas *RSPO4* was suggestively overexpressed in OM fibroblasts. In addition, *PLA2G2A* (Phospholipase A2 Group IIA), a known modulator of Wnt signaling and its directly regulated gene, was found to be strongly and specifically expressed within the OM stroma of humans [166]. Consistent with these findings, the nuclear and cytoplasmic accumulation of β-catenin in the upper layers in OM indicated the presence of nail matrix-like cells and provided further evidence for active Wnt signaling as a central driver of OM pathogenesis.

**Table 2:** Genetic and functional perturbations of Wnt signaling affecting nail mini-organ homeostasis and regeneration. This table summarizes genetic, infectious, and pharmacological perturbations of canonical Wnt signaling that affect nail mini-organ maintenance and regenerative capacity in mouse models and humans. Both loss- and gain-of-function approaches targeting Wnt ligands, receptors, intracellular mediators, and pathway modulators are included, together with associated nail phenotypes. Human genetic disorders are presented to highlight the essential and conserved role of Wnt pathway activity in nail unit morphogenesis and pathology across species.

Species/Model	Wnt Pathway Component	Type of Perturbation	Method of Manipulation	Nail Phenotype and Key Outcomes
Mouse—digit regeneration	β-catenin	Loss of function	*K14-CreER β-catenin^fx/fx^* Tam-induced epithelial-specific deletion after distal digit amputation	Loss of nail and bone regeneration; failed activation of Wnt signaling in nail Mx; impaired epithelium proliferation and osteogenic program [12]
		Gain of function	*K14-CreER β-catenin^ex3^* epithelial stabilization of β-catenin after distal digit amputation	Extended regenerative capacity due to forced epithelial Wnt activation; restoration of nail Mx signaling and bone regrowth [12]
	Wntless (WLS)	Loss of function	*K14-CreER Wls^fx/fx^* Tam-induced epithelial-specific deletion	Severely impaired nail and bone regeneration due to loss of epithelial Wnt ligand secretion β-catenin [12]
Mouse—bone homeostasis linked to nail epithelium	Canonical Wnt signaling	Functional attenuation	*K14-CreER Ctnnb1^fx/fx^* and *Wls^fx/fx^* Tam-induced epithelial-specific deletion	Distal phalanx regression and increased bone resorption caused by reduced epithelial Wnt signaling, highlighting nail epithelium–bone crosstalk [153]
Mouse—nail pathology	Canonical Wnt signaling	Functional inhibition and pharmacological treatment	Coxsackievirus A10 infection; Wnt activation with GSK3β inhibitor (CHIR99021)	Onychomadesis caused by viral suppression of Wnt signaling; pharmacological Wnt activation partially rescues nail integrity and differentiation [159]
Mouse and human	Frizzled-6 (FZD6)	Loss of function	*Fzd6* knockout mouse; biallelic *FZD6* mutations in humans	Abnormal nail formation in mice and isolated autosomal recessive nail dysplasia in humans [160,161]
Human	RSPO4	Loss of function	Homozygous or compound homozygous germline mutations	Congenital anonychia or severe hyponychia [162]
Human	RSPO4	Novel pathogenic variants	Clinical genetics—case reports	Isolated anonychia associated with additional RSPO4 variants [163]
Human	WNT10A	Loss of function	Biallelic germline mutations (OODD/ectodermal dysplasia)	Nail dystrophy or hypoplasia (odonto-onycho-dermal dysplasia spectrum) [167]
Human	WNT10A and β-catenin signaling	Functional attenuation	In vitro and tissue-based analyses of *WNT10A* variants	Reduced canonical Wnt activity [164]
Human	PORCN (Wnt ligand secretion)	Loss of function	Germline or mosaic mutation (Goltz syndrome/focal dermal hypoplasia)	Nail hypoplasia, ridging, or dysplasia due to defective Wnt ligand secretion [168]
Human	KREMEN1 (DKK1 co-receptor)	Biallelic variants—functional loss of function	Clinical genetics of ectodermal dysplasia	Ectodermal dysplasia with mild nail abnormalities [165]

## 6. Therapeutic Perspectives Targeting Wnt Signaling Pathways

Aberrant Wnt signaling has emerged as a central driver of diverse skin pathologies including its appendages—hair and nail. The evidence reveals distinct therapeutic requirements across pathology types based on whether Wnt activation or inhibition is needed. Hair loss conditions uniformly require Wnt pathway activation to restore follicular function, while fibrotic diseases and most cancers need pathway inhibition to reduce pathological proliferation. Chronic wounds present context-dependent dysregulation requiring activation in diabetic conditions but potential inhibition in keloid formation. Recent advances have led to the development of pharmacologic, epigenetic, biophysical, and regenerative strategies aimed at restoring the appropriate Wnt signaling output in skin tissues and related mini-organs.

### 6.1. Therapeutic Approaches Targeting Wnt Signaling in Skin Pathologies

In chronic non-healing wounds, Wnt signaling may be either insufficient (failing to support re-epithelialization) or aberrantly persistent, contributing to excessive matrix deposition and fibrosis. Carefully timed Wnt activation enhances healing in preclinical models, while its long-term suppression can reduce scarring—highlighting a narrow therapeutic window [6,7,24].

#### 6.1.1. Skin Fibrosis: Wnt Pathway Inhibition as an Antifibrotic Strategy

Several therapeutic strategies have been developed to inhibit pathological Wnt activation in skin fibrosis, particularly in systemic sclerosis. Small-molecule tankyrase inhibitors, including XAV-939, promote β-catenin degradation by stabilizing Axin, while β-catenin/TCF or β-catenin/CBP interaction inhibitors such as PKF118-310, ICG-001, or PRI-724 directly suppress Wnt-dependent transcription [169]. These agents demonstrated robust antifibrotic effects in preclinical models, with PRI-724 and ICG-001 showing favorable safety profiles in early-phase clinical studies.

The epigenetic reactivation of endogenous Wnt inhibitors represents an alternative approach for pathological skin fibrosis that uses DNA methyltransferase inhibitors, such as 5-aza-2′-deoxycytidine, to restore *DKK1* and *SFRP1* expression, ultimately decreasing fibroblast activation [169]. As these compounds are already clinically approved for other indications, their repurposing for fibrotic skin diseases is an attractive translational strategy. However, the long-term consequences of sustained Wnt inhibition remain unclear, particularly in tissues with a high regenerative demand, raising concerns about impaired stem cell function and delayed tissue repair. In this context, the combination therapies targeting multiple profibrotic pathways may allow lower dosing and reduce toxicity.

#### 6.1.2. Chronic Wound Healing: Therapeutic Activation of Wnt Signaling

Chronic wounds—particularly diabetic foot ulcers—in contrast to fibrosis, are characterized by insufficient canonical Wnt signaling, and therefore had to be managed by the therapeutic activation of Wnt signaling. Natural compounds, including asiaticoside and ruyi jinhuang powder, enhance Wnt-dependent transcriptional programs associated with cell proliferation and angiogenesis [23]. Stem-cell-based therapies, currently in Phase I/II trials, promote tissue regeneration partly through Wnt-mediated stem cell activation and differentiation [24].

Non-pharmacologic modalities such as photobiomodulation (PBM), extracorporeal shock wave therapy, or tetrahedral framework nucleic acids have gained attention as non-invasive strategies to activate canonical Wnt signaling in chronic wounds [24]; PBM was identified to accelerate wound closure through the stimulation of Wnt activity in HFSCs and outer root sheath cells, and the modulation of macrophage polarization. However, despite encouraging preclinical results, clinical translation remains limited by cost, accessibility, microenvironmental constraints, and the lack of standardized treatment protocols.

Interestingly, the activation of the canonical Wnt pathway through the Wnt/aPKC-modulating molecules was recently proposed as a possible disease-modifying therapy in genetic dermatoses—epidermolysis bullosa (EB) [49]. The disorder weakens the epidermal–dermal adhesion, and, although various therapeutic treatments have been already developed for the disease itself, none of these approaches strengthen this connection, which continuously results in blisters of the skin with minimal mechanical trauma. The stabilization of hemidesmosomes (HDs) through the proper activation of the canonical Wnt pathway, extrapolated into the EB treatments, might resolve this issue, as stable HDs would augment the epidermal attachment to the dermis [49]. However, it is important to recognize that excessive or prolonged Wnt activation may promote fibrotic remodeling or keloid formation, underscoring the need for tightly controlled temporal modulation.

#### 6.1.3. Skin Cancer: Wnt Pathway Inhibition as an Anticancer Strategy

Therapeutic inhibition strategies in cancer treatments target multiple levels of the Wnt pathway. Porcupine enzyme (PORCN, a membrane-bound O-acyltransferase—MBOAT which adds a palmitoylation to Wnt proteins) inhibitors, including LGK974, ETC-159, WNT-C59, IWP, CGX1321, and RXC004, block Wnt ligand secretion and have entered Phase I/II clinical trials [21,22,170], and downstream inhibitors, including tankyrase inhibitors and β-catenin transcriptional antagonists (ICG-001, PRI-724, BC2059, and CWP232228), are under active clinical development [21,22,171]. Monoclonal antibodies against Frizzled receptors (anti-FZD1/2/5/7/8), LGR5, PTK7, and ROR1, represent additional approaches [170]. In BCC, combined Hedgehog and Wnt pathway inhibition has shown enhanced efficacy, supporting pathway-crosstalk-based therapeutic strategies. For melanoma, two PORCN antagonist candidate molecules have entered clinical testing [22]. However, systemic PORCN inhibitors have also been associated with on-target toxicities in highly regenerative tissues, particularly the intestine and bone [172]. These data highlight the essential role of Wnt signaling in tissue homeostasis, and the potential risk associated with its systemic inhibition which may lead to adverse effects in normal tissues—particularly those characterized by rapid cellular turnover and regeneration dependent on the proper stem cell activation. Thus, in the context of cancer, tumor heterogeneity, context-dependent pathway function, and contradictory responses across preclinical models necessitate careful patient stratification and localized delivery approaches.

### 6.2. Therapeutic Modulation of Wnt Signaling in Pathological Hair Loss

The restoration of canonical Wnt signaling in androgenetic alopecia is a thoroughly studied approach that has already shown promising therapeutic results. This is particularly relevant in the context of a self-reinforcing DHT-PGD2 signaling loop, which suppresses Wnt/β-catenin activity via CXXC5 and GSK-3β, thereby promoting hair follicle miniaturization and limiting the regenerative capacity [25]. Accordingly, small molecules like PTD-DBM, which interfere with CXXC5, and compounds such as valproic acid and CHIR99021 were used to activate the canonical Wnt pathway [80,173,174]. The CXXC5/GSK-3β axis emerged as a key therapeutic target, with inhibitors like KY19382 being developed, demonstrating significant increases in the hair count with a favorable safety profile [25]. Small molecules that activate Wnt signaling were also identified in several natural compounds, including alpinetin, Ginkgo biloba extract, and Centipeda minima, and were described to stimulate dermal papilla cell proliferation and promote hair follicle development [89]. Furthermore, watercress extract at a 2% concentration in a six-month clinical trial improved hair thickness and density while decreasing DKK1 secretion. Several other natural products, including phytochemicals from propolis, Gardenia florida, Mangifera indica, and tectoridin, were also investigated as potential Wnt/β-catenin activators [175].

MicroRNA-based strategies offer a more targeted approach by suppressing *DKK1* expression and restoring Wnt pathway activation. Several miRNAs, including miR-29a, miR-31, miR-103/107, miR-152, and miR-218-5p, have been shown to activate Wnt-induced Frizzled-LRP5/6 complex formation and thus promote hair growth [176]. These effects were also achieved with natural compounds including morroniside, vitexin, Panax ginseng extract, and tianeptine. However, the clinical translation of miRNA therapeutics remains limited by challenges related to delivery, stability, specificity, and safety.

Regenerative medicine approaches further highlight the therapeutic relevance of Wnt signaling in hair disorders. Platelet-rich plasma (PRP), HF-derived mesenchymal stem cells, exosomes, and protein-based formulations containing Wnt ligands enhance the dermal papilla cell activity through the activation of β-catenin, ERK/Akt signaling, and anti-apoptotic pathways [25,177,178]. For example, experimental studies performed by Kandyba et al. [90] demonstrated that exogenous WNT7A is sufficient to induce the precocious telogen-to-anagen transition in mouse hair follicles, highlighting the critical role of Wnt activation in hair follicle stem cell activation and cycle initiation. These interventions have demonstrated encouraging clinical outcomes, including sustained increases in hair thickness and density, though standardization and long-term efficacy data remain limited.

Comparatively, emerging Wnt-targeted therapies, such as the topical Wnt activator SM-04554 (currently in Phase III clinical trials), show promise relative to existing FDA-approved treatments [178,179]. Nonetheless, most Wnt-modulating strategies for androgenetic alopecia remain in early-phase development. Key barriers to widespread clinical adoption include the need for standardized treatment protocols, head-to-head comparisons with established therapies, and a long-term safety assessment, particularly given the pathway’s central role in stem cell and cancer biology.

#### Therapy-Induced Hair Loss

Therapy-induced hair loss resulting from genotoxic cancer treatments such as chemotherapy and radiotherapy is a common and distressing complication for which effective preventive strategies remain limited [180]. The experimental work in a mouse model by Huang et al. provided robust experimental evidence directly linking Wnt pathway disruption to hair loss, demonstrating that ionizing radiation suppresses canonical Wnt signaling in a dose- and P53-dependent manner [181]. The activation of the DNA damage response regulator P53 inhibits proliferative signaling pathways, including the Wnt activity in hair follicles, ultimately contributing to follicular dystrophy and alopecia. Importantly, the authors showed that the augmentation of Wnt signaling, through the stabilization of β-catenin with the GSK3β inhibitor, restored ectopic progenitor proliferation and prevented both radiation- and cyclophosphamide-induced alopecia [181]. In a different experimental strategy, a mouse model of cyclophosphamide-induced alopecia was treated with a collagen-targeted parathyroid hormone analog (PTH-CBD), in order to promote canonical Wnt activity [182]. The treatment increased the number of anagen VI follicles and improved cyclophosphamide-associated hair loss. These findings suggest that impaired Wnt signaling may represent a convergent mechanism contributing to hair loss across different cytotoxic modalities.

However, other signaling pathways, including Shh, EGFR, and TRAIL-mediated apoptosis, have also been implicated in chemotherapy-induced follicle damage, indicating that therapy-induced alopecia results from the disruption of multiple regulatory nodes controlling hair follicle cycling and survival [183,184,185]. Consistent with this view, studies using a human hair follicle xenograft model of alkylating chemotherapy showed that permanent alopecia reflects the loss of regenerative competence following busulfan/cyclophosphamide exposure, accompanied by the depletion of stemness-associated programs, including a reduced Fzd1 expression, although PI3K/Akt-driven stem-cell mobilization followed by P53/P38-dependent apoptosis appeared to represent the dominant mechanism rather than Wnt suppression alone [186]. Importantly, attempts to enhance this regenerative axis remain largely preclinical, and no Wnt-targeted therapy is currently included in routine clinical management, which is still dominated by supportive approaches such as scalp cooling rather than pathway-direct intervention.

### 6.3. Opportunities in Nail and Digit Enhanced Regeneration

The mammalian nail mini-organ is positioned as a powerful model for understanding Wnt-dependent epithelial–mesenchymal interactions, the neural–epithelial connection, and stem-cell regulation in an appendage capable of natural regeneration. Moreover, the transition between regeneration and fibrosis occurs across an exceptionally narrow anatomical window, enabling the direct comparison of these opposing outcomes within the same tissue context. These insights, together with the accessibility of the tissue, underscore its value as an experimental platform for Wnt-targeted regenerative therapies.

However, regardless of numerous experiments being performed on such a remarkable model, no clinically applicable treatments currently exist for modulating the Wnt pathway in nail or digit regeneration. To date, only gene-based experimental approaches have been reported, in which *β-catenin* and *Lgr6* were used as direct therapeutic targets to manipulate the Wnt pathway activity in the nail epithelium [26]. These studies were performed on mice models with the conditional knockout or overexpression of *β-catenin* in the epithelium, which were subjected to digit tip amputation prior to treatment. The results demonstrated that the activation of Wnt signaling via β-catenin or the modulation of *Lgr6* can influence regenerative responses.

The therapeutic manipulation of Wnt in the nail mini-organ remains largely unexplored clinically, but represents a promising direction. Multiple studies highlight significant opportunities to enhance nail regeneration through the targeted manipulation of Wnt signaling, while underscoring the substantial mechanistic gaps in fully understanding how this pathway regulates regenerative processes. Thus far, *Lgr6* and *β-catenin* have emerged as promising therapeutic targets, with combination strategies and the precise temporal control of pathway activation proposed as critical determinants of regenerative outcomes.

Future therapeutic strategies may benefit from integrating Wnt-modulating biomaterials with a better understanding of how mechanical forces from the NP influence regeneration, while also tailoring treatments to a patient’s individual Wnt activity levels. For example, the local delivery of Wnt agonists or biomaterials that mimic Wnt signals at the amputation site might help extend the regenerative response beyond the distal phalanx. At the same time, expanding nail stem cells ex vivo under carefully controlled Wnt conditions could enable the development of grafts for nail bed reconstruction or combined nail–bone repair [12,13,149]. However, currently, the lack of clinically translatable approaches and a limited understanding of the dose- and time-dependent effects of Wnt modulation remain significant barriers to therapeutic development.

### 6.4. Pathway Crosstalk and Future Therapeutic Directions

Wnt signaling operates within a complex signaling network, interacting with the TGF-β, Notch, BMP, and Hedgehog pathways [24]. In fibrosis and wound healing, dysregulated crosstalk amplifies pathological outcomes, while, in hair follicle biology, balanced Wnt–BMP–Shh interactions are required for tissue homeostasis [80]. These insights highlight the need for multitarget therapeutic strategies rather than single-pathway modulation.

Emerging approaches, including CRISPR-based gene editing, stem-cell–biomaterial integration, and targeted small-molecule modulators, offer new avenues to fine-tune Wnt signaling with a greater specificity [80]. Combination therapies addressing hormonal, inflammatory, oxidative, and signaling abnormalities may be particularly effective in complex disorders such as androgenetic alopecia.

Despite the rapid progress, key barriers remain, including a long-term safety assessment, delivery challenges, cost, and the preservation of stem cell compartments. Addressing these challenges will be critical for translating Wnt-targeted therapies into durable and accessible clinical solutions.

### 6.5. Limitations and Challenges of Wnt-Targeted Therapies

Despite the substantial advances in Wnt-targeted therapeutic strategies, several critical limitations constrain their clinical translation. A fundamental challenge arises from the pleiotropic and context-dependent nature of Wnt signaling, which plays essential roles in stem cell maintenance, tissue homeostasis, and regeneration across multiple organs. Consequently, both the systemic activation and inhibition of the Wnt pathway carry substantial risks, including tumorigenesis, impaired tissue renewal, or fibrosis. In this context, the narrow therapeutic window associated with Wnt modulation is another key limitation. While transient activation may promote regeneration, prolonged or excessive signaling can lead to aberrant proliferation or pathological remodeling, particularly in fibrotic conditions and cancer. Conversely, sustained Wnt inhibition may compromise stem cell function and delay tissue repair, particularly in high-turnover tissues such as the intestine, skin, and bone.

Technical and translational barriers are additional challenges for treatment delivery. The Wnt pathway has historically been considered difficult to target pharmacologically due to the lack of conventional druggable nodes, while RNA-based and gene-editing approaches face delivery, stability, and safety constraints. And, especially in skin, the tissue-specific targeting is of the utmost importance, as different compartments (epidermal, hair follicle, and nail units) exhibit distinct Wnt signaling dynamics.

Therapeutic efficacy is further limited by pathway redundancy and compensatory signaling. Extensive crosstalk between Wnt and other signaling pathways, including TGF-β, Notch, BMP, and Hedgehog, complicate therapeutic interventions through adaptive responses that could attenuate treatment effects or promote resistance, as exemplified by the WNT5A-mediated resistance mechanism in melanoma or self-reinforcing signaling loops in androgenetic alopecia. Targeting Wnt signaling in isolation may therefore be insufficient, supporting the need for combinatorial or multi-pathway approaches. Moreover, the existence of multiple endogenous modulators, including DKKs, SFRPs, and R-spondins, create a buffered signaling network that is difficult to modulate with single-target interventions. However, regardless of the delivery route, therapy timing, and target, most Wnt-signaling approaches remain in preclinical or early-phase clinical development, with limited long-term high-quality data, and inconsistent results across model systems, reflecting the differences in the tissue architecture, immune context, and disease chronicity.

Collectively, effective interventions must account for the distinct roles of Wnt signaling across skin compartments and disease states, as well as its dynamic functions during different phases of regeneration and repair. These considerations highlight the need for localized, temporally controlled, and combinatorial therapeutic strategies, to maximize the therapeutic benefit while minimizing the adverse effects of Wnt-targeted therapies.

## 7. Summary

Wnt signaling constitutes a fundamental regulatory axis governing homeostasis and regeneration across the adult skin and its mini-organs: hair follicles and the nails. Although unified by a shared molecular architecture, Wnt signaling operates in a highly context-dependent and organ-specific manner: it tightly balances repair versus fibrosis in wounded skin, drives stem cell activation and cycling in hair follicles, and coordinates epithelial–mesenchymal interactions during nail and digit regeneration. Perturbations in this finely tuned system lead to profound pathological outcomes ranging from hair loss and chronic wounds to fibrosis and cancer. Importantly, therapeutic strategies must therefore be tailored to the biological context—requiring activation in degenerative or regenerative failure states and inhibition in hyperproliferative or fibrotic conditions. Advances in pharmacologic modulators, regenerative medicine, biomaterials, and gene-based approaches have opened promising translational avenues; however, precise temporal control, pathway crosstalk integration, and long-term safety remain critical challenges. A deeper mechanistic understanding of Wnt dynamics within each cutaneous mini-organ will be essential for developing targeted, safe, and durable therapeutic interventions.

## Data Availability

No new data were created or analyzed in this study. Data sharing is not applicable to this article.
